# Nickel-catalysed cross-electrophile coupling of aryl bromides and primary alkyl bromides†

**DOI:** 10.1039/d2ra00010e

**Published:** 2022-01-26

**Authors:** Nanxing Gao, Yanshun Li, Dawei Teng

**Affiliations:** State Key Laboratory Base of Eco-Chemical Engineering, College of Chemical Engineering, Qingdao University of Science and Technology Qingdao 266042 China dteng@qust.edu.cn

## Abstract

The structure of primary alkylated arenes plays an important role in the molecular action of drugs and natural products. The nickel/spiro-bidentate-pyox catalysed cross-electrophile coupling of aryl bromides and primary alkyl bromides was developed for the formation of the Csp^2^–Csp^3^ bond, which provided an efficient method for the synthesis of primary alkylated arenes. The reactions could tolerate functional groups such as ester, aldehyde, ketone, ether, benzyl, and imide.

## Abstract 2

The structure of primary alkylated arenes play an important role in the molecular action of drugs and natural products. The nickel/spiro-bidentate-pyox catalysed cross-electrophile coupling of aryl bromides and primary alkyl bromides was developed.

## Graphical abstract

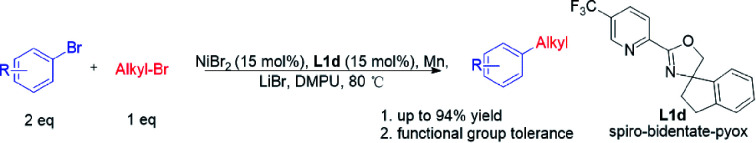

## Introduction

**Scheme 1:**
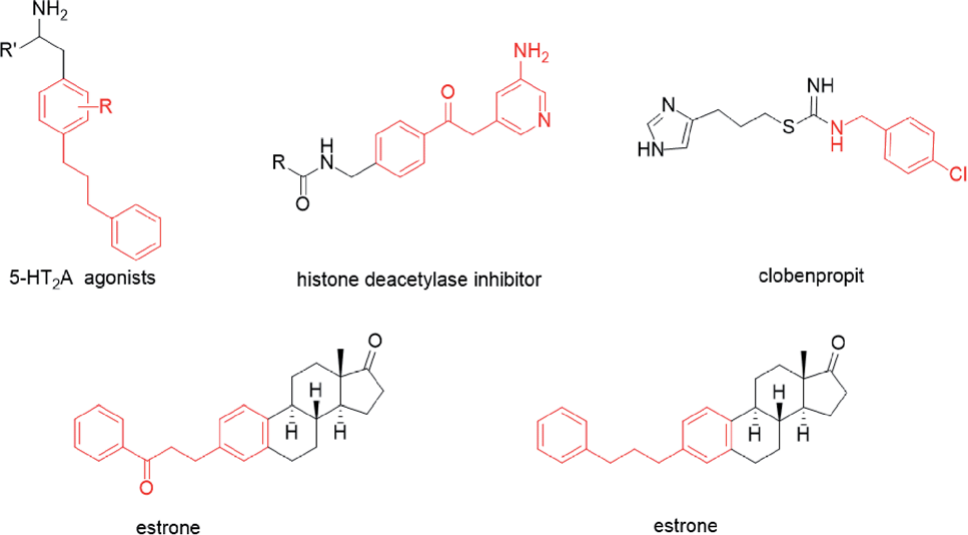
Drugs with the structure of alkylated arenes.

Numerous attractive synthesis approaches to primary alkylated arenes have been developed, primarily based on the transition metal-catalysed addition of nucleophiles to electrophiles.^1^ Cross-electrophile coupling represents an important field in these modern organic synthesis reactions.^2^ In general, the direct use of electrophiles is convenient because electrophiles are more accessible and easier to use than nucleophiles in the coupling reactions. The cross electrophile coupling reactions catalysed by nickel metal catalysts can provide effective methods for the construction of new C–C bonds in drugs and natural products.^2*b*,3^ Previous studies have established that 5-HT_2_A agonists have been implicated in cardiovascular function^4^ (Scheme 1). A class of histone deacetylase inhibitors has been generated from *N*-(2-amino-4-pyridyl)benzamide derivatives, which could be applied in the treatment of cancer, leukemia, and diseases related to differentiation and proliferation.^5^ Clobenpropit, a histamine H_3_-receptor antagonist, shows good activity *in vitro* at subnanomolar concentrations.^6^ Additionally, there are other alkylated alkanes derived from biologically important molecules such as estrones.^7^ Therefore, the development of a tremendous cross-electrophile coupling reaction between alkyl bromides and aryl bromides is still widely sought.

**Scheme 2:**
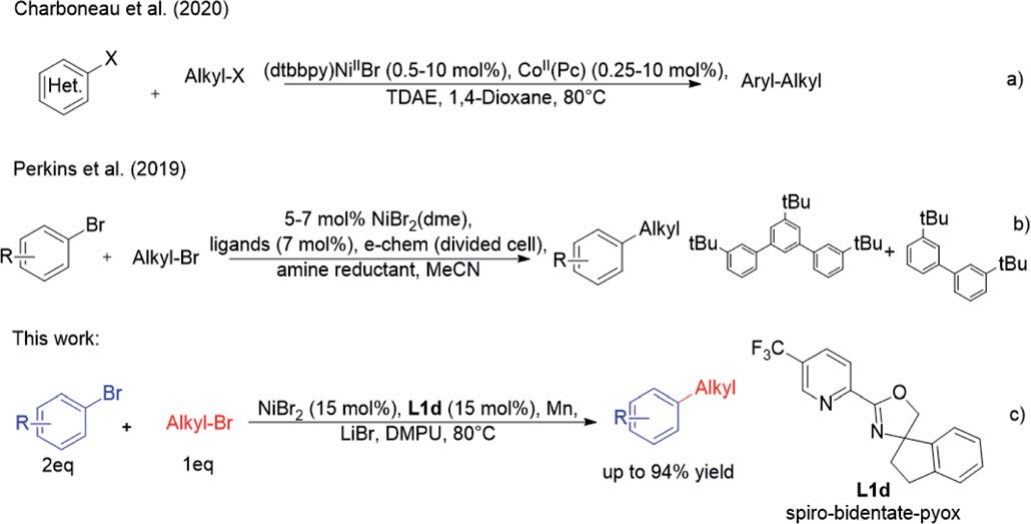
Ni-catalysed cross-electrophile coupling between aryl bromides and primary alkyl bromides.

Recently, Charboneau *et al.*^8^ used a dual metal catalytic system for cross-electrophile coupling reactions between aryl halides and alkyl halides (Scheme 2a). Perkins *et al.*^9^ demonstrated that the cross-electrophile coupling reaction catalysed by nickel catalyst could be achieved under metal-reductant-free electrochemical conditions (Scheme 2b). As part of an effort to develop nickel-catalysed cross-electrophile coupling reactions, we previously achieved a direct cross-electrophile coupling of cyclic secondary alkyl bromides with aryl bromides.^10^ Owing to the good catalytic effect of spiro-bidentate-pyox ligands, we reasoned that they may also have a good catalytic effect in other types of cross-electrophile coupling reactions, which could lead to alkylated arenes (Scheme 2c).

## Results and discussion

To identify whether the primary alkyl bromides and aryl bromides were competent, the coupling reaction of 4-bromobenzoate 1a with 1-bromo-3-phenylpropane 2a was surveyed (Table 1). Initial tests of nickel salts as metal catalysts for the cross-electrophile couplings were carried out using manganese as the reductant and *N*-methylpyrrolidone (NMP) as the solvent. Good catalytic performance was achieved using NiBr_2_ as the metal catalyst (entry 1). When NiBr_2_ was replaced by NiI_2_, NiBr_2_·glyme, or NiCl_2_, the yield of the desired cross-coupling product 3a decreased (entries 2–4). The desired product was not detected when the organic nickel catalyst Ni(acac)_2_ was used (entry 5). In order to optimize the reaction with different structures of the catalysts, we synthesized ligands with a substituted group on the pyridyl and phenyl ring L1b–L1d. Using NiBr_2_ as the metal catalyst, ligand L1d with an electron-withdrawing group at the C5-position on the pyridine ring gave the cross-coupling product 3a in 72% yield, which is superior to the ligands L1a and L1c with electron-neutral or electron-donating groups as well as ligand L1b with a substituent group on the phenyl ring (entries 6–8). Other bidentate ligands, like L2–L5, did not yield better results (entries 9–12). Decreasing the temperature from 80 °C to 60 °C produced 3a only in 39% yield (entry 13). Raising the temperature to 100 °C also resulted in a dramatic decrease in the yield (entry 14). In addition, no desired product was detected without NiBr_2_ or L1d, which implied that the metal and ligand were indispensable for the coupling reactions (entries 15–16).

**Table 1:** Scope of metal catalysts, ligands, and temperature^a^^,^^b^

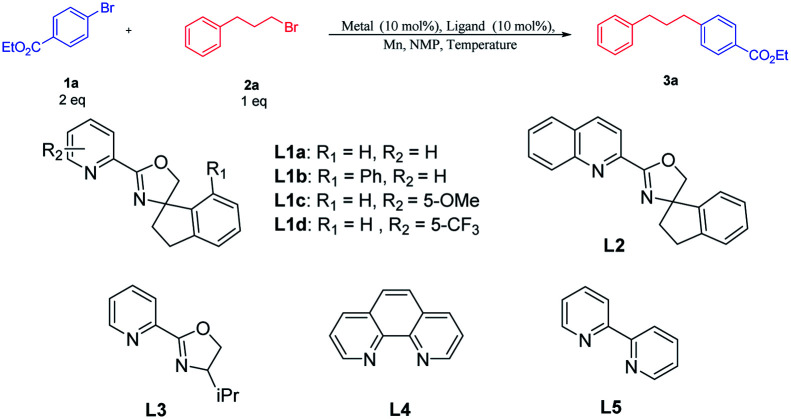				
Entry	Metal	Ligand	Temperature	Yield (%)
1	NiBr_2_	L1a	80 °C	67
2	NiI_2_	L1a	80 °C	62
3	NiBr_2_·glyme	L1a	80 °C	56
4	NiCl_2_	L1a	80 °C	Trace
5	Ni(acac)_2_	L1a	80 °C	NR^c^
6	NiBr_2_	L1b	80 °C	35
7	NiBr_2_	L1c	80 °C	25
8	NiBr_2_	L1d	80 °C	72
9	NiBr_2_	L2	80 °C	Trace
10	NiBr_2_	L3	80 °C	10
11	NiBr_2_	L4	80 °C	55
12	NiBr_2_	L5	80 °C	35
13	NiBr_2_	L1d	60 °C	49
14	NiBr_2_	L1d	100 °C	39
15	—	L1d	80 °C	0
16	NiBr_2_	—	80 °C	0

a Reaction conditions: 1a (0.40 mmol), 2a (0.20 mmol), Mn (0.60 mmol), ligand (0.02 mmol), metal (0.02 mmol), NMP (1 mL).

b Isolated yield.

c No reaction.

Following the evaluation of the metal catalysts, ligands, and temperatures, other reaction conditions were screened. Among the amide solvents,^11^ NMP can reach 72% yield under the reaction conditions (Table 2, entry 1). When *N*,*N*-dimethylformamide (DMF) and *N*,*N*-dimethylacetamide (DMA) were used as solvents, the yields were both decreased (entries 2 and 3). 1,3-Dimethyltetrahydropyrimidin-2(1*H*)-one (DMPU) proved to be the best solvent; the yield could reach 76% (entry 4). Catalyst loading screening found that the yield of 3a was promoted to 84% when the amounts of NiBr_2_ and L1d were both increased to 15 mol% (entry 6), but the yield of 3a decreased when the loading of the metal and ligand were increased to 20 mol% or decreased to 7.5 mol% (entries 5 and 7). Further optimization found that the use of zinc instead of manganese as a reductant led to low yield (entry 8). The addition of one equivalent of lithium bromide boosted the yield to 91%;^12^ however, lithium chloride was not as beneficial as we previously observed,^10^ and other additives did not generate better yields (entries 9–12). The screening of different loading of lithium bromide did not furnish a better result (entries 13–14).

**Table 2:** Optimization of reaction conditions^a^^,^^b^

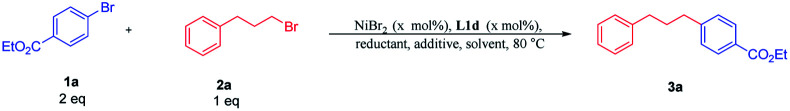					
Entry	Solvent	Additive	Temperature	Reductant	Yield (%)
1	NMP	—	80 °C	Mn	72
2	DMF	—	80 °C	Mn	40
3	DMA	—	80 °C	Mn	66
4	DMPU	—	80 °C	Mn	76
5^c^	DMPU	—	80 °C	Mn	78
6^d^	DMPU	—	80 °C	Mn	84
7^e^	DMPU	—	80 °C	Mn	80
8	DMPU	—	80 °C	Zn	54
9	DMPU	LiCl	80 °C	Mn	61
10	DMPU	LiBr	80 °C	Mn	91
11	DMPU	NaI	80 °C	Mn	77
12	DMPU	MgCl_2_	80 °C	Mn	26
13^f^	DMPU	LiBr	80 °C	Mn	80
14^g^	DMPU	LiBr	80 °C	Mn	90

a Reaction conditions: 1a (0.40 mmol), 2a (0.20 mmol), additive (0.20 mmol), reductant (0.60 mmol), L1d (0.02 mmol), NiBr_2_ (0.02 mmol), solvent (1 mL).

b Isolated yield.

c *x* = 7.5.

d *x* = 15.

e *x* = 20.

f Additive (0.15 mmol).

g Additive (0.30 mmol).

Using the optimal reaction conditions, a range of substituted aryl bromides 1 were examined for the coupling reaction with 2a, furnishing a series of alkylated arenes 3 (Table 3). The challenging steric-hindered aryl bromide substrates 1b are reasonably tolerated, and the coupling product 3b was obtained in a slightly low yield. Additionally, aryl bromide with ester substituent at the *meta*-position of the benzene ring was obtained in moderate yield, providing the product 3c in the yield of 52%. Among the functional groups, the reactions were successfully observed in the presence of esters 3a–d, naphthyl 3e, aldehyde 3f, and ketones 3g, 3h. It should be noted that 3f is the intermediate product of 5-HT_2_A.^4*c*^ The electron-rich substrate 3i could also be coupled with 2a in good yield. However, the reaction was not effective for aryl bromides bearing electron-rich reagents like 4-bromoanisole.

**Table 3:** Scope of aryl bromides in XEC^a^^,^^b^

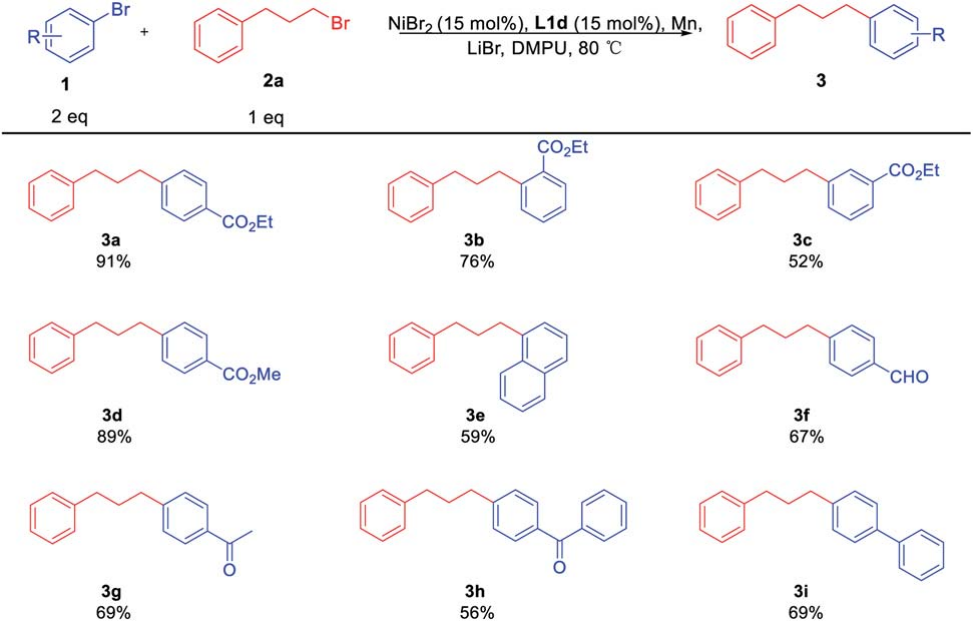

a Reaction conditions: 1 (0.40 mmol), 2a (0.20 mmol), LiBr (0.20 mmol), Mn (0.60 mmol), L1d (0.03 mmol), NiBr_2_ (0.03 mmol), DMPU (1 mL).

b Isolated yield.

Then, the scope of the reaction with respect to primary alkyl bromides 2 was subsequently investigated. The results were shown in Table 4. All reactions provide the alkylated cross-coupling products smoothly. The functional groups, such as the ether 3j and benzyloxy 3h, all worked efficiently under the standard conditions. This catalytic method was also compatible with nitrogen-containing electrophiles, as demonstrated by the coupling of 2-(1-bromopropan-2-yl)isoindoline-1,3-dione 2l and *N*-(2-bromoethyl)phthalimide 2m with 1a in 61% and 62% yield, respectively. In the synthesis of the relatively longer-chain product 3n, it was found that the coupling product was obtained in satisfactory yield. Besides, a simple shorter-chain alkyl substrate 2o was converted to 3o smoothly. These results further showed that it did not significantly influence the reaction performance when changing the alkyl chains.

**Table 4:** Scope of alkyl bromides in XEC^a^^,^^b^

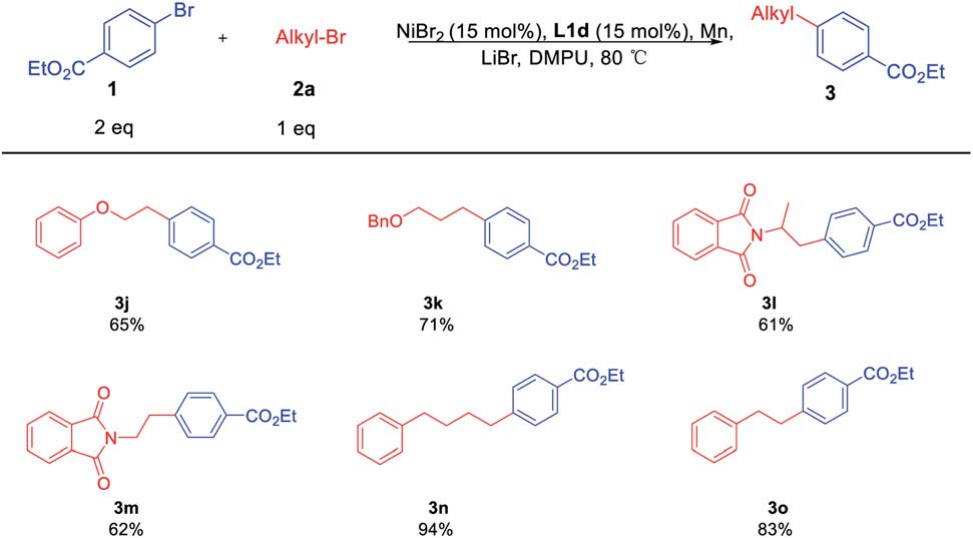

a Reaction conditions: 1a (0.40 mmol), 2 (0.20 mmol), LiBr (0.20 mmol), Mn (0.60 mmol), L1d (0.03 mmol), NiBr_2_ (0.03 mmol), DMPU (1 mL).

b Isolated yield.

**Scheme 3:**
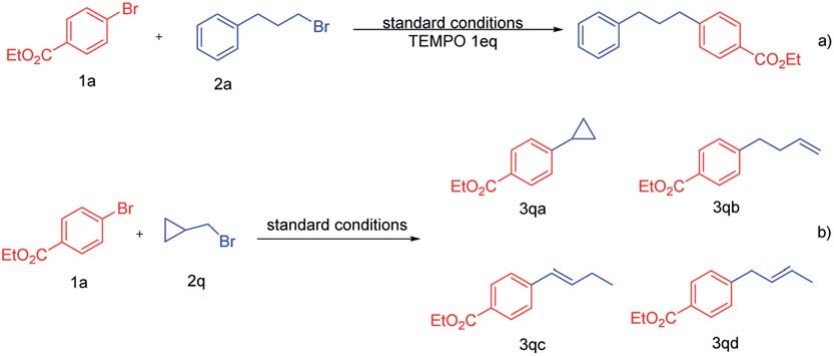
Mechanistic studies.

To further understand the mechanism of this reaction, the radical experiment was subsequently carried out (Scheme 3). Under the standard conditions, in the presence of the radical scavenger 2,2,6,6-tetramethyl-1-piperidinyloxy (TEMPO), the cross-coupling reaction was completely blocked and no alkylated arene 3a was recorded, which implies that this transformation might undergo a free radical pathway (Scheme 3a). To further demonstrate that the alkyl bromide might follow the radical pathway, a radical clock experiment was carried out.^13,12*d*^ We used the cyclopropylmethyl bromide 2q as a coupling reagent under the standard conditions, and the cross-coupling product 3qa was not detected (Scheme 3b). The reaction resulted in a mixture of products; it was found that the reaction generates the ring-opened products 3qb, 3qc and 3qd in a total 51% yield, and the ratio of 3qb : 3qd : 3qe is about 2 : 0.4 : 2.4. These results further point towards the involvement of alkyl radical intermediate generation in the coupling process of alkyl bromides.

**Scheme 4:**
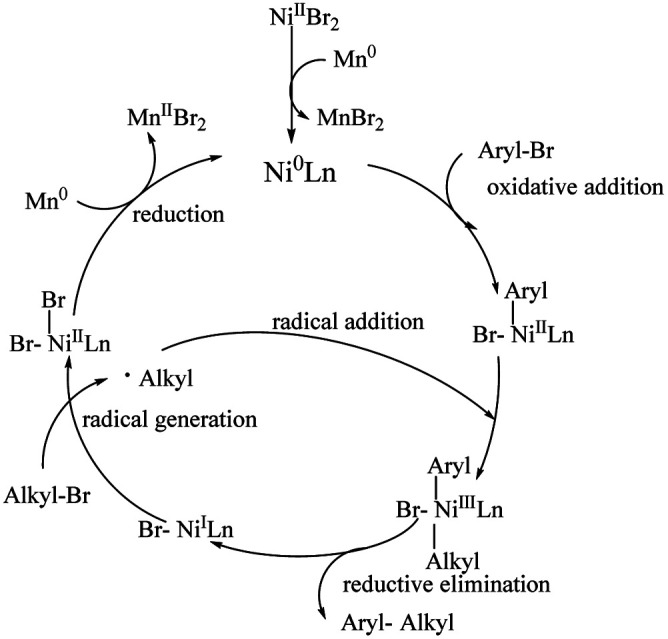
The plausible mechanism.

The current mechanistic hypothesis is outlined in Scheme 4. The reaction begins with the initial reduction of the Ni^II^ precatalyst, which can furnish a catalytically active Ni^0^Ln species. Subsequent oxidative addition to the aryl bromide forms an aryl-Ni^II^ intermediate. Then, it reacts with an alkyl radical to give an aryl alkyl Ni^III^ intermediate. Reductive elimination occurs to give the aryl–alkyl cross-coupling product along with generating the Ni^I^ species. The radical generation gives the Ni^II^ intermediate, which is subjected to reduction by manganese and regenerates the Ni^0^Ln species to complete the catalytic cycle.

## Conclusions

In conclusion, by using a NiBr_2_/L1d catalyst, the cross-electrophile coupling reactions of aryl bromides and primary alkyl bromides were developed. A variety of alkylated arene products with various functional groups could be obtained in moderate to excellent yields. The result further demonstrated that the nickel/spiro-bidentate-pyox catalytic cross-electrophile coupling protocol afforded an effective method for the synthesis of alkylated arene products. Further investigation to extend this catalytic protocol to other coupling reactions and a detailed mechanistic study are ongoing and will be reported in due course.

## Conflicts of interest

There are no conflicts to declare.

## Supplementary Material

RA-012-D2RA00010E-s001
