# Supplementary material for: Nickel-catalysed cross-electrophile coupling of aryl bromides and primary alkyl bromides
Source: RSC Adv. 2022 Jan 26;12(6):3569–72. doi: 10.1039/d2ra00010e (PMC8979266; doi:10.1039/d2ra00010e)

Electronic Supplementary Material (ESI) for RSC Advances.
This journal is © The Royal Society of Chemistry 2022

Supporting information

Nickel-Catalyzed Cross-Electrophile Coupling of Aryl Bromides and Primary Alkyl Bromides with Spiro-bidentate-pyox Ligand

Nanxing Gao, Yanshun Li and Dawei Teng*

State Key Laboratory Base of Eco-Chemical Engineering, College of Chemical Engineering, Qingdao University of Science and Technology, Qingdao 266042, China

Table of Contents

I. Experimental Section **1**

General 1

The Procedure for Cross-Electronphile Coupling product **3** 1

Characterization data of compound **3** 2

Radical Clock Experiment 6

II. Spectral Data for Compounds **3** 7

*Corresponding author. E-mail addresses: dteng@qust.edu.cn

I. Experimental Section

General

All reagents were purchased from commercial suppliers and used without purification. All reactions were monitored by TLC. Chromatography refers to open column chromatography on silica gel (200-300 mesh). Melting points were uncorrected values. ^{1}H NMR spectra were recorded at 500 MHz and ^{13}C NMR spectra were recorded at 126 MHz by using a Bruker Avance 500M spectrometer. Mass spectra were performed on an Ultima Global spectrometer with an ESI source.

General procedure for the cross electrophile coupling product 3

Details of Optimization and Control Experiments 3

A typical procedure for optimization and control reactions: To a dried vial equipped with a magnetic stir bar was added metal catalyst (x mmol) and additive (if applicable) in glove box. The vial was moved out of the glove box, ligand and solvent were added via syringe. The reaction mixture was stirred for one hour at room temperature. Aryl bromide (0.40 mmol), alkyl bromide (0.20 mmol) and reductant (0.60 mmol) were added sequentially. The reaction mixture was allowed to stir under argon atmosphere at heated. After the reaction finished, it was directly loaded onto a silica column without work-up. Through flash column chromatography (SiO_2: ethyl acetate in petroleum) to provide the cross coupling product.

The Procedure for Cross-Electronphile Coupling product 3

To a dried vial equipped with a magnetic stir bar was added $NiBr_2$ (6.6 mg, 0.03 mmol) and lithium bromide (17.4 mg, 0.20 mmol) in glove box. The vial was moved out of the glove box, **L1d** (9.60 mg, 0.03 mmol) and DMPU (1 mL) were added via syringe. The reaction mixture was stirred for one hour at room temperature. Aryl bromide (0.40 mmol), alkyl bromide (0.20 mmol) and manganese powder (33 mg, 0.60 mmol) were added sequentially. The reaction mixture was allowed to stir under argon atmosphere at 80 ºC. After the reaction finished, it was directly loaded onto a silica column without work-up. Through flash column chromatography (SiO_2: ethyl acetate in petroleum) to provide the cross coupling product.

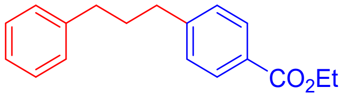

Ethyl 4-(3-phenylpropyl)benzoate **3a**

The compound was reported previously in *Angew. Chem. Int. Ed*, **2018**, 57, 11339-11343.

The title compound was isolated as a colorless oil in 91% yield.

^{1}H NMR (500 MHz, Chloroform-d) δ 8.02 (d, J = 8.2 Hz, 2H), 7.34 (t, J = 7.5 Hz, 2H), 7.30 (d, J = 7.8 Hz, 2H), 7.27 – 7.21 (m, 3H), 4.42 (q, J = 7.1 Hz, 2H), 2.75 (t, J = 7.7 Hz, 2H), 2.70 (t, J = 7.7 Hz, 2H), 2.07 – 1.98 (m, 2H), 1.44 (t, J = 7.1 Hz, 3H). ^{13}C NMR (126 MHz, Chloroform-d) δ 166.82, 147.83, 142.06, 129.78, 128.55, 128.49, 128.29, 125.99, 60.90, 35.54, 35.46, 32.73, 14.48. HRMS (ESI-TOF^{+}) $[M+H]^+$ m/z calcd for $C_{18}H_{20}O_2H^+$ 269.1542, found 269.1544.

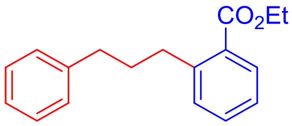

Ethyl 2-(3-phenylpropyl)benzoate **3b**

The compound was reported previously in *Nature Chemistry*, **2016**, 8, 1126-1130.

The title compound was isolated as a colorless oil in 76% yield.

^{1}H NMR (500 MHz, Chloroform-d) δ 7.89 (dd, J = 7.7, 1.5 Hz, 1H), 7.44 (td, J = 7.5, 1.5 Hz, 1H), 7.34 – 7.25 (m, 5H), 7.25 – 7.19 (m, 3H), 4.37 (q, J = 7.1 Hz, 2H), 3.06 – 3.01 (m, 2H), 2.73 (t, J = 7.8 Hz, 2H), 2.01 – 1.94 (m, 2H), 1.41 (t, J = 7.1 Hz, 3H). ^{13}C NMR (126 MHz, Chloroform-*d*) δ 168.03, 144.00, 142.50, 131.85, 131.00, 130.72, 130.16, 128.57, 128.41, 125.96, 125.85, 60.95, 36.07, 34.30, 33.53, 14.46. HRMS (ESI-TOF^{+}) $[M+H]^+$ m/z calcd for $C_{18}H_{20}O_2H^+$ 269.1542, found 269.1542.

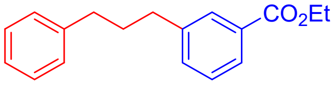

Ethyl 3-(3-phenylpropyl)benzoate **3c**

The compound was reported previously in *Nature Chemistry*, **2009**, 1, 494-499.

The title compound was isolated as a colorless oil in 52% yield.

^{1}H NMR (500 MHz, Chloroform-d) δ 7.91 – 7.86 (m, 2H), 7.40 – 7.33 (m, 2H), 7.30 (dd, J = 8.7, 6.5 Hz, 2H), 7.20 (dd, J = 7.7, 5.6 Hz, 3H), 4.39 (q, J = 7.2 Hz, 2H), 2.69 (dt, J = 21.3, 7.7 Hz, 4H), 2.03 – 1.95 (m, 2H), 1.41 (t, J = 7.1 Hz, 3H). ^{13}C NMR (126 MHz, Chloroform-d) δ 166.95, 142.67, 142.15, 133.12, 130.67, 129.63, 128.57, 128.48, 128.43, 127.20, 125.96, 61.04, 35.51, 35.33, 32.98, 14.49. HRMS (ESI-TOF^{+}) $[M+H]^+$ m/z calcd for $C_{18}H_{20}O_2H^+$ 269.1542, found 269.1545.

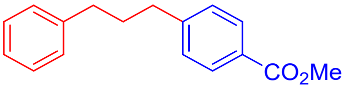

Methyl 4-(3-phenylpropyl)benzoate **3d**

The compound was reported previously in *ACS Catalysis*, **2020**, 10, 12642-12656

The title compound was isolated as a colorless oil in 89% yield.

^{1}H NMR (500 MHz, Chloroform-d) δ 8.00 (d, J = 8.0 Hz, 2H), 7.33 (t, J = 7.5 Hz, 2H), 7.30 – 7.27 (m, 2H), 7.26 – 7.20 (m, 3H), 3.94 (s, 3H), 2.74 (t, J = 7.7 Hz, 2H), 2.69 (t, J = 7.7 Hz, 2H), 2.05 – 1.98 (m, 2H). ^{13}C NMR (126 MHz, Chloroform-*d*) δ 167.30, 147.97, 142.04, 129.82, 128.60, 128.55, 128.50, 127.93, 126.00, 52.10, 35.54, 35.47, 32.72. HRMS (ESI-TOF$^+$) $[M+Na]^+$ m/z calcd for $C_{17}H_{18}O_2Na^+$ 277.1204, found 277.1215.

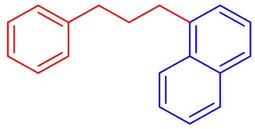

1-(3-Phenylpropyl)naphthalene **3e**

The compound was reported previously in *Organic Letters*, **2018**, 20, 5644-5647.

The title compound was isolated as a colorless oil in 59% yield.

^{1}H NMR (500 MHz, Chloroform-*d*) δ 8.04 – 7.99 (m, 1H), 7.94 – 7.87 (m, 1H), 7.77 (d, *J* = 8.1 Hz, 1H), 7.53 (tt, *J* = 7.1, 5.1 Hz, 2H), 7.45 (dd, *J* = 8.2, 7.0 Hz, 1H), 7.40 – 7.33 (m, 3H), 7.30 – 7.23 (m, 3H), 3.20 – 3.13 (m, 2H), 2.82 (t, *J* = 7.7 Hz, 2H), 2.20 – 2.12 (m, 2H). ^{13}C NMR (126 MHz, Chloroform-*d*) δ 142.32, 138.54, 134.03, 132.01, 128.89, 128.62, 128.47, 126.70, 126.05, 125.95, 125.83, 125.65, 125.53, 123.93, 36.02, 32.68, 32.39. HRMS (ESI-TOF$^+$) $[M+Na]^+$ m/z calcd for $C_{19}H_{18}Na^+$ 269.1306, found 269.1299.

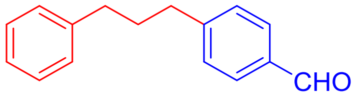

4-(3-Phenylpropyl)benzaldehyde **3f**

The compound was reported previously in *ACS Catalysis*, **2020**, 10, 12642-12656.

The title compound was isolated as a colorless oil in 67% yield.

^{1}H NMR (500 MHz, Chloroform-d) δ 9.98 (s, 1H), 7.81 (d, J = 7.8 Hz, 2H), 7.35 (d, J = 7.8 Hz, 2H), 7.30 (t, J = 7.5 Hz, 2H), 7.23 – 7.17 (m, 3H), 2.74 (t, J = 7.8 Hz, 2H), 2.67 (t, J = 7.7 Hz, 2H), 2.00 (tt, J = 9.1, 6.8 Hz, 2H). ^{13}C NMR (126 MHz, Chloroform-d) δ 192.12, 149.94, 141.89, 134.66, 130.07, 129.24, 128.53, 126.06, 35.73, 35.48, 32.64. HRMS (ESI-TOF$^+$) $[M+H]^+$ m/z calcd for $C_{16}H_{16}OH^+$ 225.1279, found 225.1277.

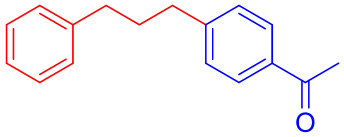

1-(4-(3-Phenylpropyl)phenyl)ethan-1-one **3g**

The compound was reported previously in *J. Am. Chem. Soc.* **2016**, 138, 5016−5019.

The title compound was isolated as a colorless oil in 69% yield.

^{1}H NMR (500 MHz, Chloroform-d) δ 7.90 (d, J = 7.9 Hz, 2H), 7.29 (dq, J = 10.4, 6.0, 4.4 Hz, 4H), 7.20 (dd, J = 10.8, 7.4 Hz, 3H), 2.72 (t, J = 7.8 Hz, 2H), 2.67 (t, J = 7.7 Hz, 2H), 2.60 (s, 3H), 1.99 (tt, J = 8.8, 6.9 Hz, 2H). ^{13}C NMR (126 MHz, Chloroform-d) δ 197.99, 148.28, 141.99, 135.20, 128.77, 128.65, 128.54, 128.50, 126.01, 35.52, 35.47, 32.67, 26.67. HRMS (ESI-TOF$^+$) $[M+H]^+$ m/z calcd for $C_{17}H_{18}OH^+$ 239.1436, found 239.1434.

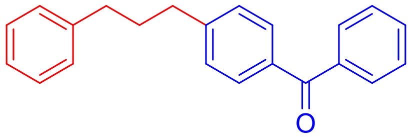

Phenyl(4-(3-phenylpropyl)phenyl)methanone **3h**

The compound was reported previously in *Organic Letters*, 2020, 22, 2350-2353.

The title compound was isolated as a colorless oil in 56% yield.

^{1}H NMR (500 MHz, Chloroform-*d*) δ 7.62 – 7.56 (m, 2H), 7.52 (dd, *J* = 8.4, 2.1 Hz, 2H), 7.43 (dd, *J* = 8.5, 6.8 Hz, 2H), 7.37 – 7.24 (m, 6H), 7.23 – 7.18 (m, 3H), 2.70 (td, *J* = 7.7, 4.2 Hz, 4H), 2.01 (tdd, *J* = 9.3, 6.9, 4.1 Hz, 2H). ^{13}C NMR (126 MHz, Chloroform-*d*) δ 142.39, 141.56, 141.26, 138.85, 129.00, 128.85, 128.60, 128.46, 127.19, 127.13, 125.91, 35.60, 35.19, 33.05. HRMS (ESI-TOF^{+}) [M+H]$^{+}$ m/z calcd for $C_{22}H_{20}OH^{+}$ 301.1592, found 301.1570.

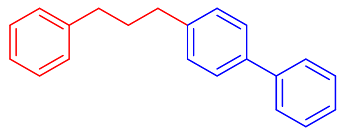

4-(3-Phenylpropyl)-1,1'-biphenyl **3i**

The compound was reported previously in *Organic Letters*, **2020**, 22, 2350-2353.

The title compound was isolated as a colorless oil in 69% yield.

^{1}H NMR (500 MHz, Chloroform-*d*) δ 7.59 (dt, *J* = 8.4, 1.9 Hz, 2H), 7.55 – 7.50 (m, 2H), 7.43 (dd, *J* = 8.5, 6.8 Hz, 2H), 7.37 – 7.24 (m, 6H), 7.23 – 7.18 (m, 3H), 2.70 (td, *J* = 7.7, 4.2 Hz, 4H), 2.05 – 1.96 (m, 2H). ^{13}C NMR (126 MHz, Chloroform-*d*) δ 142.39, 141.56, 141.26, 138.85, 129.00, 128.85, 128.60, 128.46, 127.19, 127.13, 125.91, 35.60, 35.19, 33.05. HRMS (ESI-TOF^{+}) [M+H]$^{+}$ m/z calcd for $C_{21}H_{20}H^{+}$ 273.1643, found 273.1652; [M+Na]$^{+}$ m/z calcd for $C_{21}H_{20}Na^{+}$ 295.1463, found 295.1479.

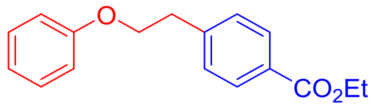

Ethyl 4-(2-phenoxyethyl)benzoate **3j**

The title compound was isolated as a colorless oil in 65% yield.

^{1}H NMR (500 MHz, Chloroform-d) δ 7.97 (d, J = 8.1 Hz, 2H), 7.33 (d, J = 8.0 Hz, 2H), 7.26 – 7.21 (m, 2H), 6.91 (t, J = 7.3 Hz, 1H), 6.88 – 6.83 (m, 2H), 4.34 (q, J = 7.1 Hz, 2H), 4.17 (t, J = 6.8 Hz, 2H), 3.12 (t, J = 6.8 Hz, 2H), 1.36 (t, J = 7.1 Hz, 3H). ^{13}C NMR (126 MHz, Chloroform-d) δ 166.66, 158.75, 143.86, 129.85, 129.59, 129.10, 128.94, 121.01, 114.68, 68.08, 60.97, 35.90, 14.46. HRMS (ESI-TOF^{+}) [M+H]$^{+}$ m/z calcd for $C_{17}H_{18}O_{3}H^{+}$ 271.1334, found 271.1339.

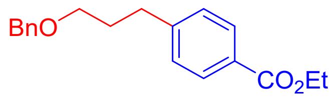

Ethyl 4-(3-(benzyloxy)propyl)benzoate **3k**

The title compound was isolated as a colorless oil in 71% yield.

^{1}H NMR (500 MHz, Chloroform-*d*) δ 7.96 (d, *J* = 8.3 Hz, 2H), 7.35 (d, *J* = 5.9 Hz, 4H), 7.30 (td, *J* = 5.9, 3.4 Hz, 1H), 7.24 (d, *J* = 8.1 Hz, 2H), 4.51 (s, 2H), 4.37 (q, *J* = 7.1 Hz, 2H), 3.48 (t, *J* = 6.3 Hz, 2H), 2.81 – 2.75 (m, 2H), 1.99 – 1.91 (m, 2H), 1.39 (t, *J* = 7.1 Hz, 3H). ^{13}C NMR (126 MHz, Chloroform-*d*) δ 166.81, 147.57, 138.58, 129.78, 128.61, 128.52, 128.29, 127.81, 127.73, 73.11, 69.32, 60.90, 32.56, 31.19, 14.49. HRMS (ESI-TOF^{+}) [M+Na]$^{+}$ m/z calcd for $C_{19}H_{22}O_{3}Na^{+}$ 321.1467, found 321.1454.

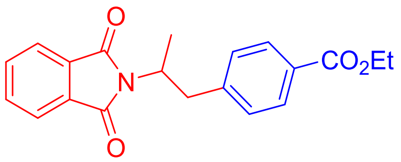

Ethyl 4-(2-(1,3-dioxoisoindolin-2-yl)propyl)benzoate **3l**

The title compound was isolated as a colorless oil in 61% yield.

^{1}H NMR (500 MHz, Chloroform-d) δ 7.87 (dd, J = 8.2, 2.5 Hz, 2H), 7.78 – 7.70 (m, 2H), 7.65 (td, J = 5.0, 2.8 Hz, 2H), 7.24 (dd, J = 8.2, 2.2 Hz, 2H), 4.67 (tdd, J = 11.7, 6.9, 3.5 Hz, 1H), 4.31 (qd, J = 7.0, 2.8 Hz, 2H), 3.42 (ddd, J = 13.1, 9.8, 2.1 Hz, 1H), 3.13 (ddd, J = 13.9, 6.4, 2.3 Hz, 1H), 1.54 (dd, J = 7.0, 2.2 Hz, 3H), 1.34 (td, J = 7.1, 3.3 Hz, 3H). ^{13}C NMR (126 MHz, Chloroform-*d)* δ 168.40, 166.62, 143.81, 134.00, 131.82, 129.86, 129.00, 128.93, 123.22, 60.95, 48.40, 39.92, 18.63, 14.42. HRMS (ESI-TOF$^+$) [M+Na]$^+$ m/z calcd for $C_{20}H_{19}NO_4Na^+$ 360.1212, found 360.1211.

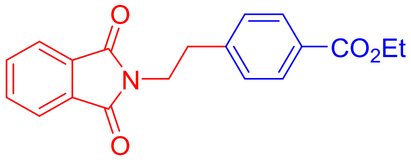

Ethyl 4-(2-(1,3-dioxoisoindolin-2-yl)ethyl)benzoate **3m**

The title compound was isolated as a white solid in 62% yield.

^{1}H NMR (500 MHz, Chloroform-d) δ 7.94 (d, J = 8.0 Hz, 2H), 7.81 (dt, J = 7.5, 3.7 Hz, 2H), 7.70 (dd, J = 5.4, 3.0 Hz, 2H), 7.30 (d, J = 8.0 Hz, 2H), 4.34 (q, J = 7.1 Hz, 2H), 3.94 (dd, J = 8.4, 6.7 Hz, 2H), 3.05 (t, J = 7.6 Hz, 2H), 1.37 (t, J = 7.1 Hz, 3H). ^{13}C NMR (126 MHz, Chloroform-d) δ 168.21, 166.60, 143.36, 134.12, 132.08, 129.97, 129.11, 128.97, 123.41, 60.99, 38.93, 34.65, 14.44. Melting Point: 115-116 °C. HRMS (ESI-TOF$^+$) [M+Na]$^+$ m/z calcd for $C_{19}H_{17}NO_4\ Na^+$ 346.1055, found 346.1049.

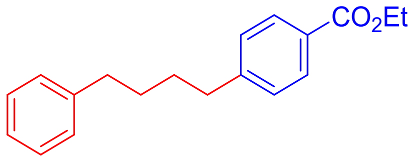

Ethyl 4-(4-phenylbutyl)benzoate **3n**

The compound was reported previously in *Org. Biomol. Chem*. **2016***,* 14*,* 3314-3321.

The title compound was isolated as a colorless oil in 94% yield.

^{1}H NMR (500 MHz, Chloroform-d) δ 7.97 (d, J = 8.1 Hz, 2H), 7.29 (t, J = 7.5 Hz, 2H), 7.24 (d, J = 7.9 Hz, 2H), 7.21 – 7.15 (m, 3H), 4.38 (q, J = 7.1 Hz, 2H), 2.70 (t, J = 7.0 Hz, 2H), 2.65 (t, J = 7.0 Hz, 2H), 1.69 (dt, J = 7.2, 3.4 Hz, 4H), 1.40 (t, J = 7.1 Hz, 3H). ^{13}C NMR (126 MHz, Chloroform-d) δ 166.82, 148.09, 142.47, 129.74, 128.51, 128.42, 128.21, 125.85, 60.88, 35.94, 35.86, 31.09, 30.81, 14.48. HRMS (ESI-TOF$^+$) [M+H]$^+$ m/z calcd for $C_{19}H_{22}O_2H^+$ 283.1698, found 283.1694.

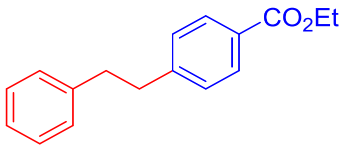

Ethyl 4-phenethylbenzoate **3o**

The compound was reported previously in *Organic Letters*, **2010**, 12, 1692-1695.

The title compound was isolated as a colorless oil in 83% yield.

^{1}H NMR (500 MHz, Chloroform-d) δ 8.03 (d, J = 8.2 Hz, 2H), 7.39 – 7.32 (m, 2H), 7.29 (t, J = 7.9 Hz, 3H), 7.25 – 7.20 (m, 2H), 4.44 (q, J = 7.1 Hz, 2H), 3.10 – 2.96 (m, 4H), 1.47 (t, J = 7.1 Hz, 3H). ^{13}C NMR (126 MHz, Chloroform-d) δ 166.68, 147.05, 141.17, 129.66, 128.50, 128.46, 128.40, 128.30, 126.08, 60.81, 37.88, 37.49, 14.36. HRMS (ESI-TOF^{+}) $[M+H]^{+}$ m/z calcd for $C_{17}H_{18}O_{2}H^{+}$ 255.1385, found 255.1377.

Radical Clock Experiment

To a dried vial equipped with a magnetic stir bar was added $NiBr_2$ (6.6 mg, 0.03 mmol) and lithium bromide (17.4 mg, 0.20 mmol) in glove box. The vial was moved out of the glove box, **L1d** (9.60 mg, 0.03 mmol) and DMPU (1 mL) were added via syringe. The reaction mixture was stirred for one hour at room temperature. Then **1a** (0.40 mmol), cyclopropylmethyl bromide (0.20 mmol) and manganese powder (33 mg, 0.60 mmol) were added sequentially. The reaction mixture was allowed to stir under argon atmosphere at 80 ºC. After the reaction finished, it was directly loaded onto a silica column without work-up. Through flash column chromatography (SiO_2: ethyl acetate in petroleum) to provide the ring-opened products **3qb-3qd** in 51% yield, the ratio of the **3qb**: **3qd**: **3qe** is about 2:0.4: 2.4. The ratio of the products was determined by ^{1}H NMR (500 MHz) analysis (Figure S1).

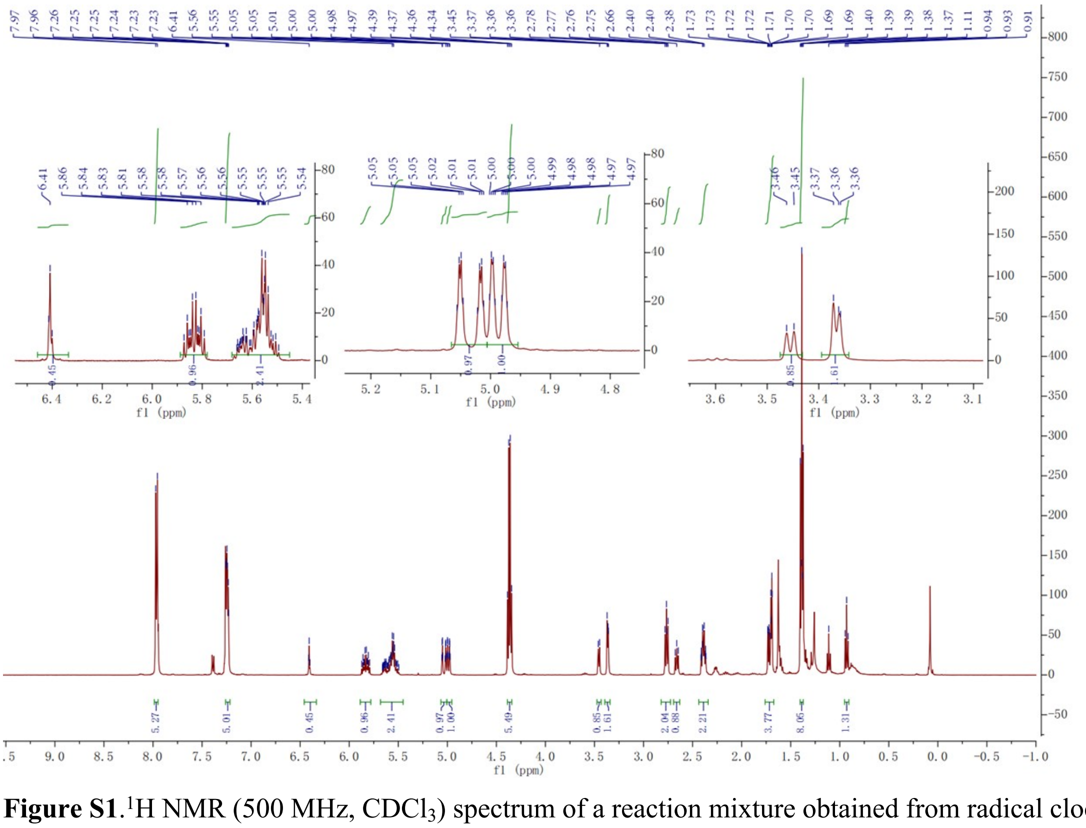

Figure S1.^{1}H NMR (500 MHz, $CDCl_3$) spectrum of a reaction mixture obtained from radical clock experiment

II. Spectral Data for Compounds 3

^{1}H NMR spectra of ethyl 4-(3-phenylpropyl)benzoate 3a

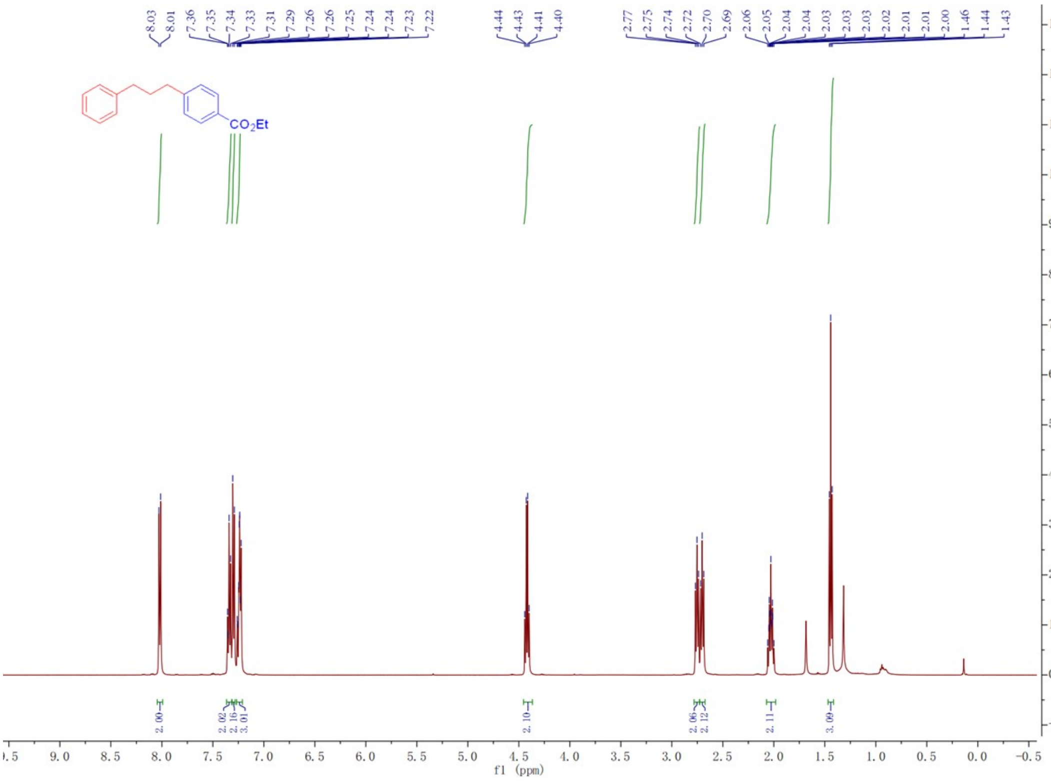

^{13}C NMR spectra of ethyl 4-(3-phenylpropyl)benzoate 3a

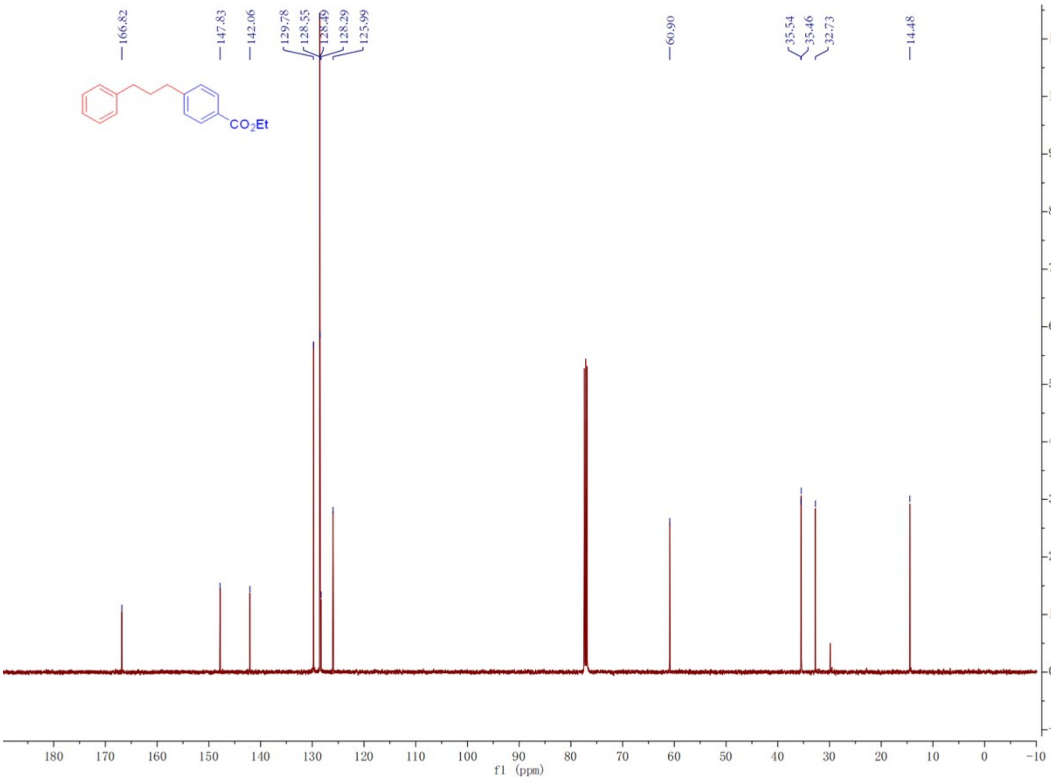

^{1}H NMR spectra of ethyl 2-(3-phenylpropyl)benzoate 3b

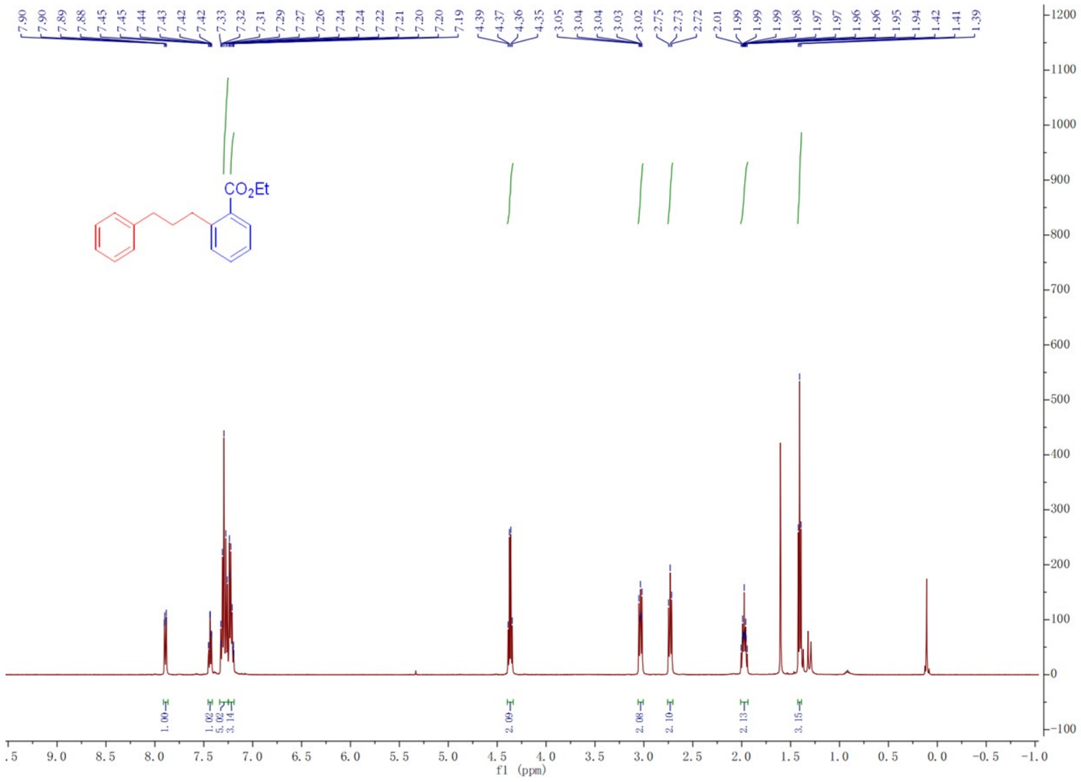

^{13}C NMR spectra of ethyl 2-(3-phenylpropyl)benzoate 3b

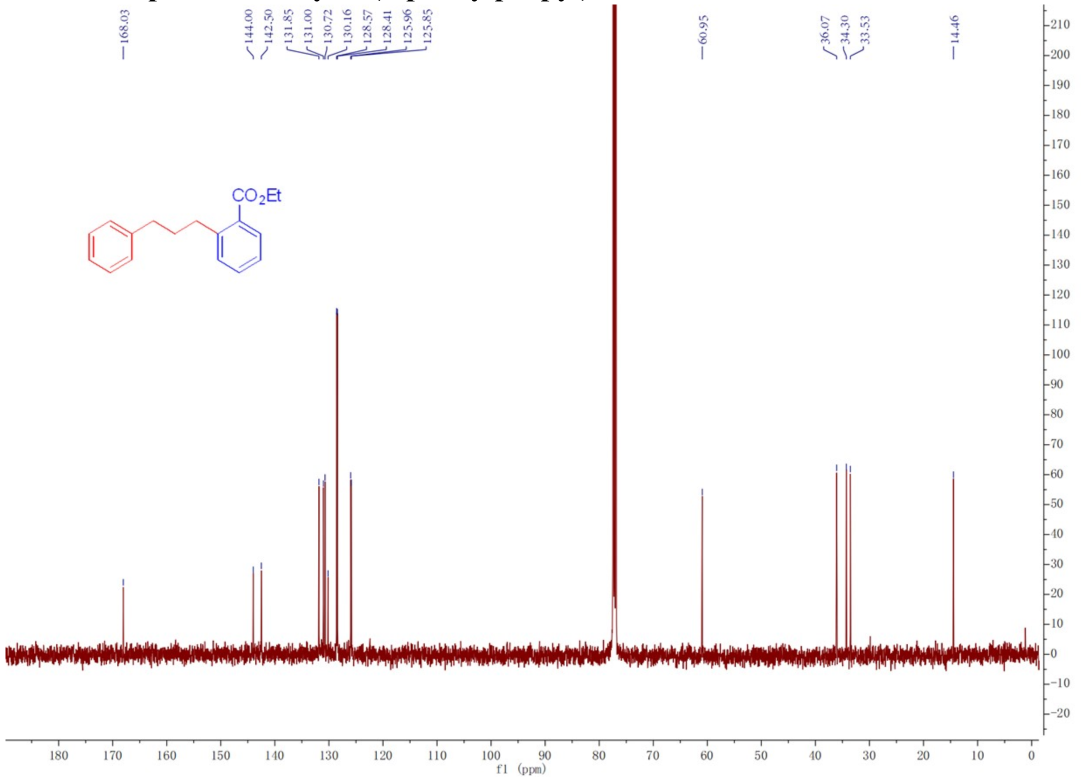

^{1}H NMR spectra of ethyl 3-(3-phenylpropyl)benzoate 3c

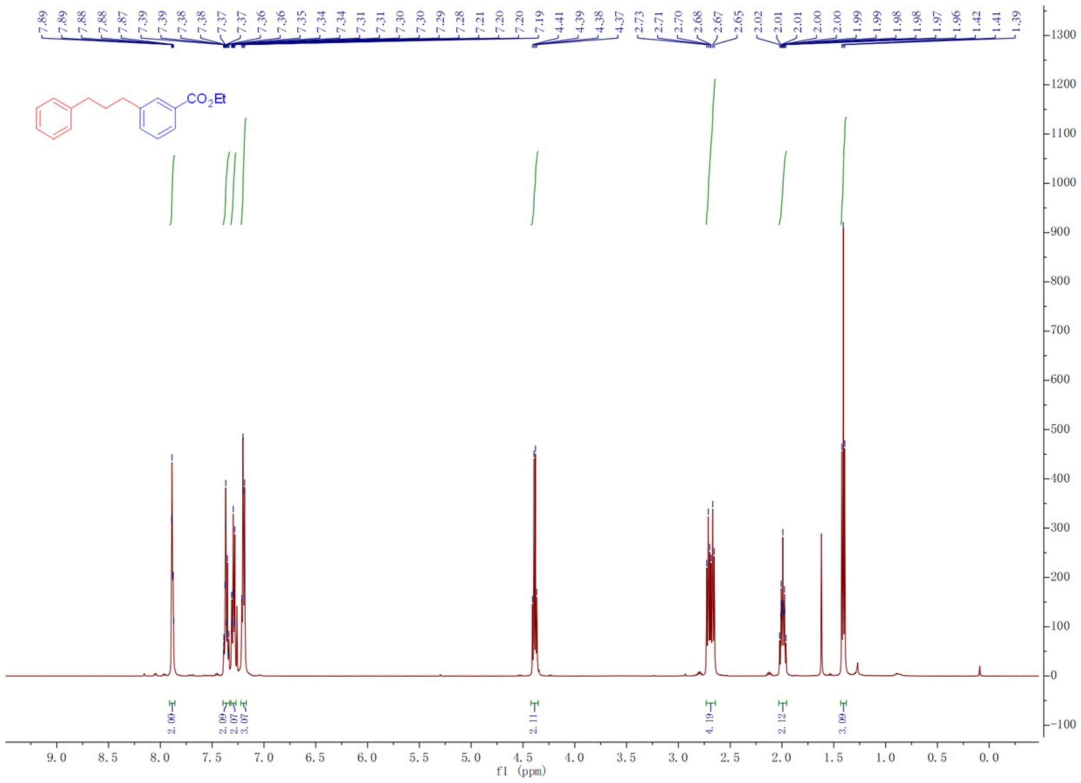

^{13}C NMR spectra of ethyl 3-(3-phenylpropyl)benzoate 3c

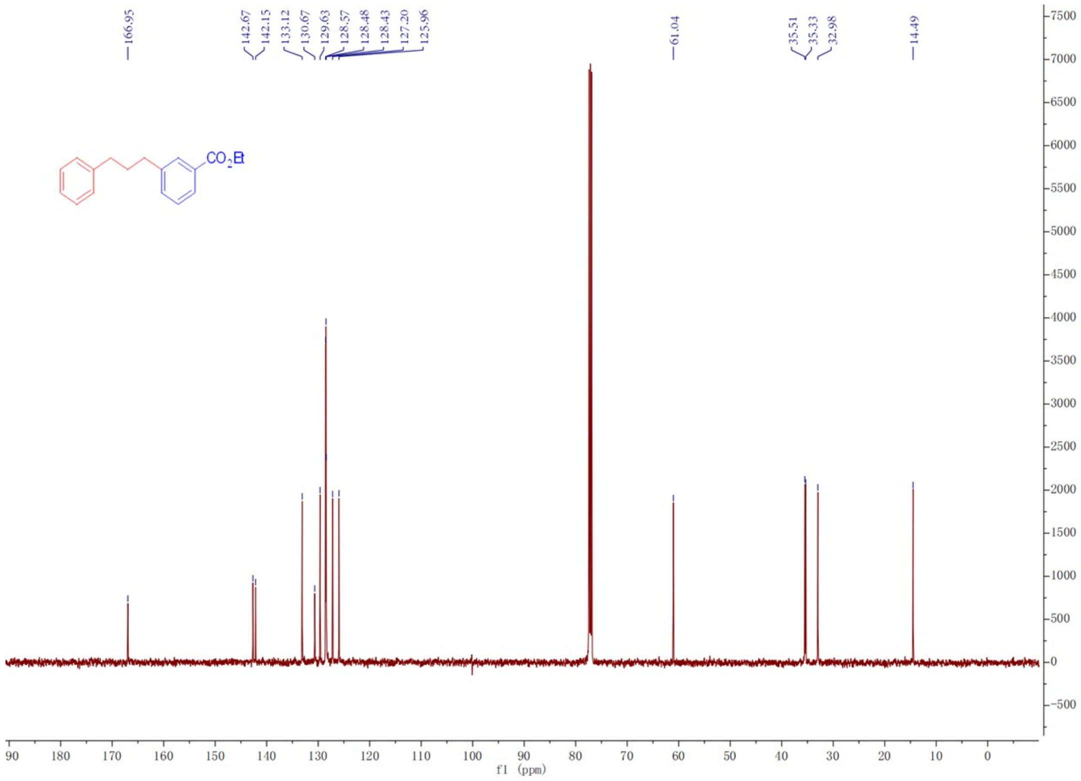

^{1}H NMR spectra of methyl 4-(3-phenylpropyl)benzoate 3d

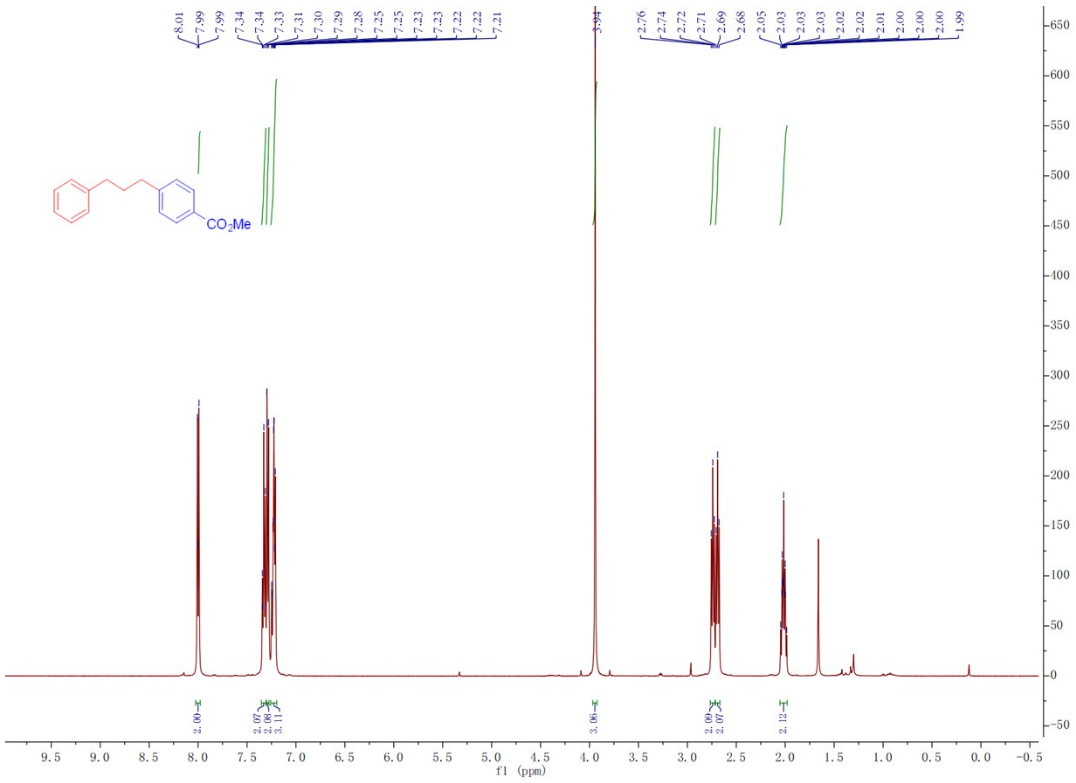

^{13}C NMR spectra of methyl 4-(3-phenylpropyl)benzoate 3d

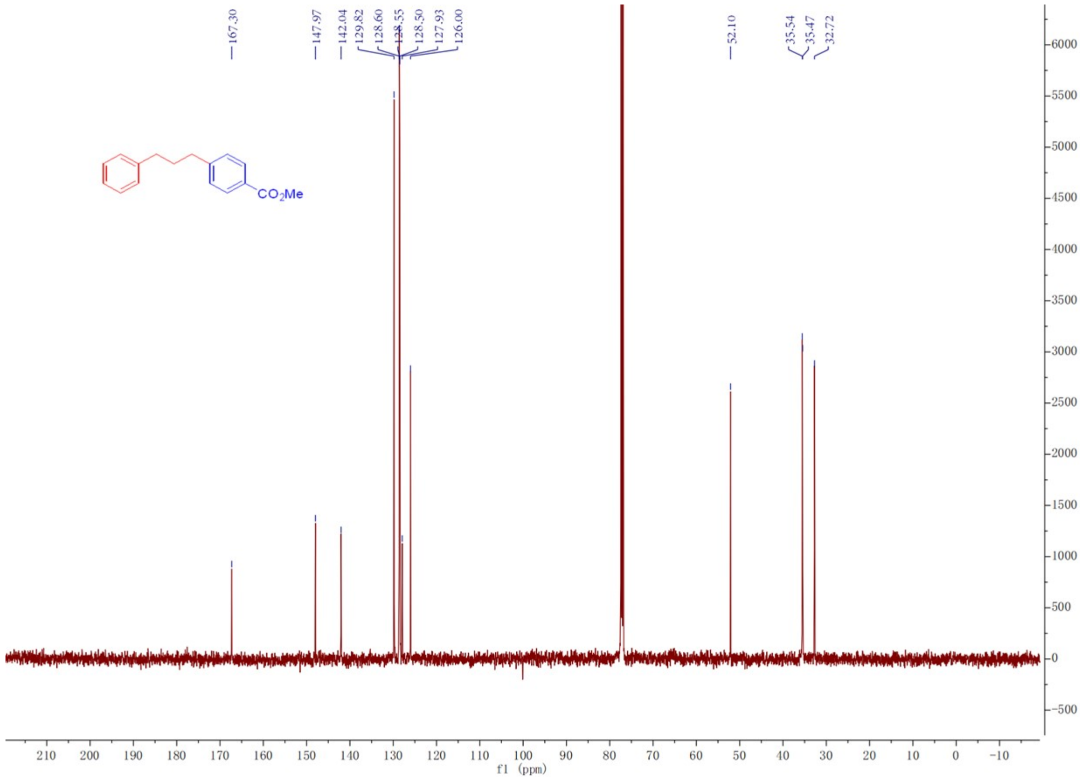

^{1}H NMR spectra of 1-(3-phenylpropyl)naphthalene 3e

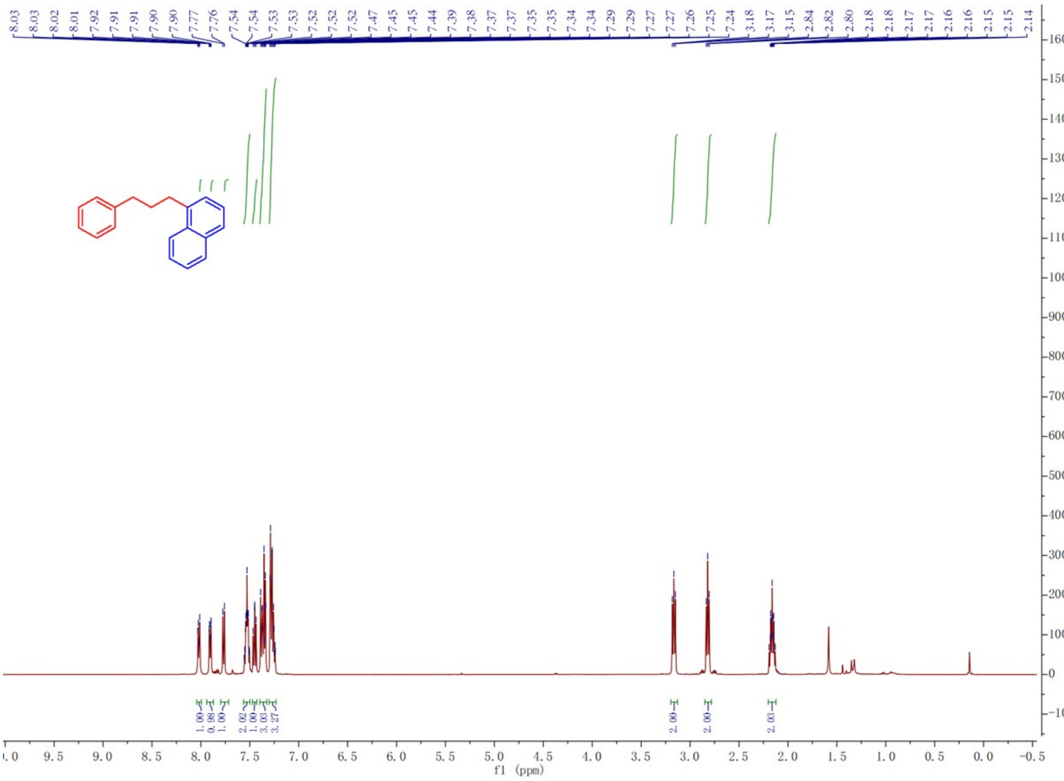

^{13}C NMR spectra of 1-(3-phenylpropyl)naphthalene 3e

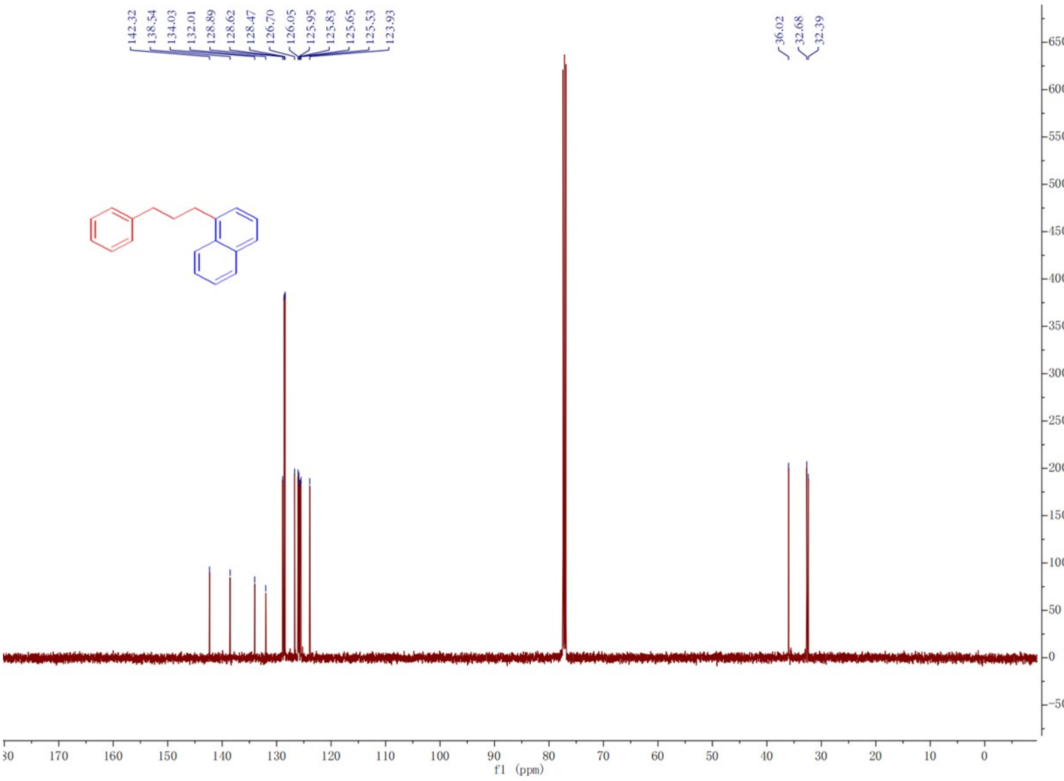

^{1}H NMR spectra of 4-(3-phenylpropyl)benzaldehyde 3f

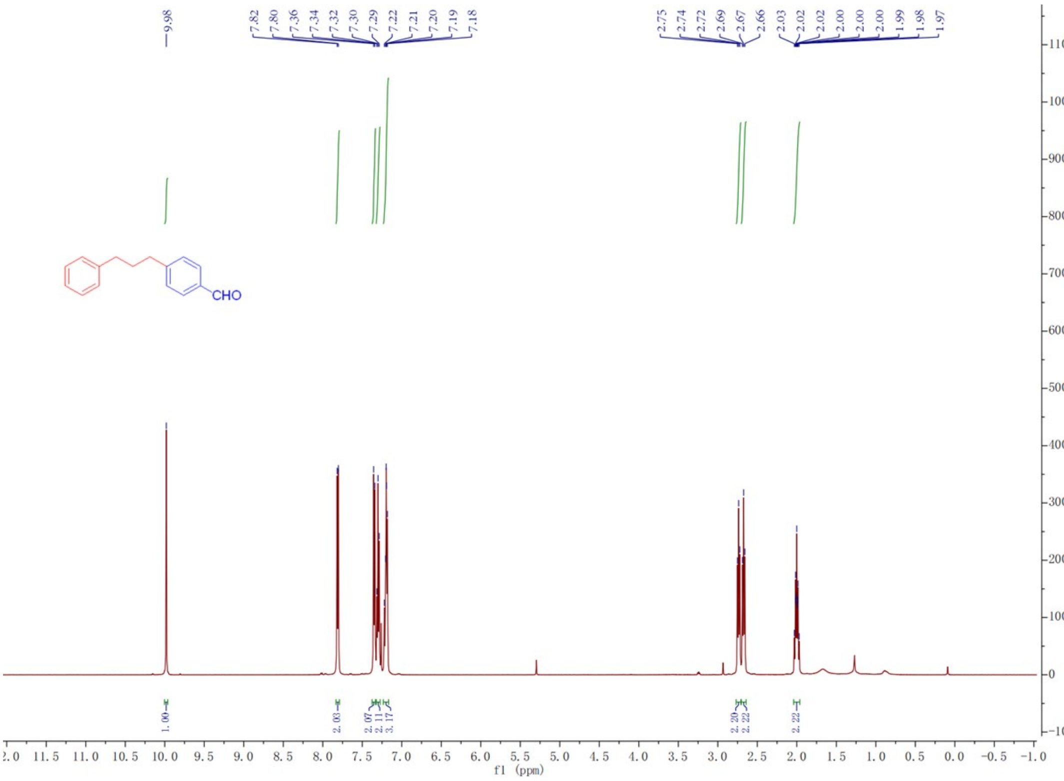

^{13}C NMR spectra of 4-(3-phenylpropyl)benzaldehyde 3f

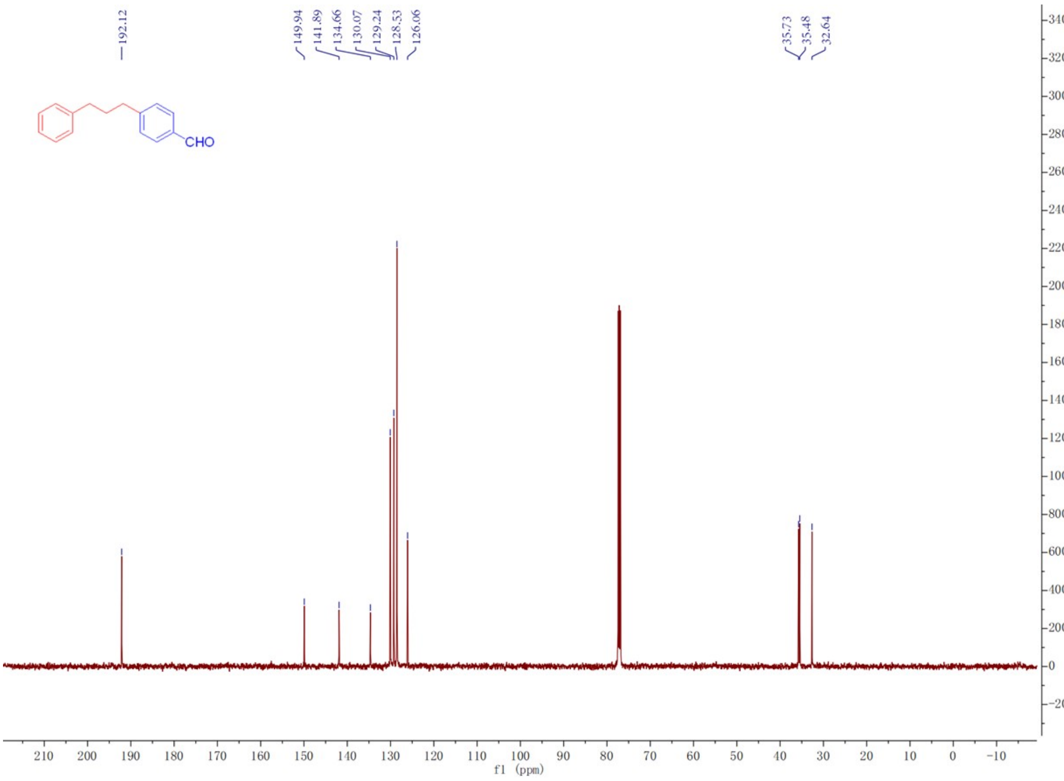

^{1}H NMR spectra of 1-(4-(3-phenylpropyl)phenyl)ethan-1-one 3g

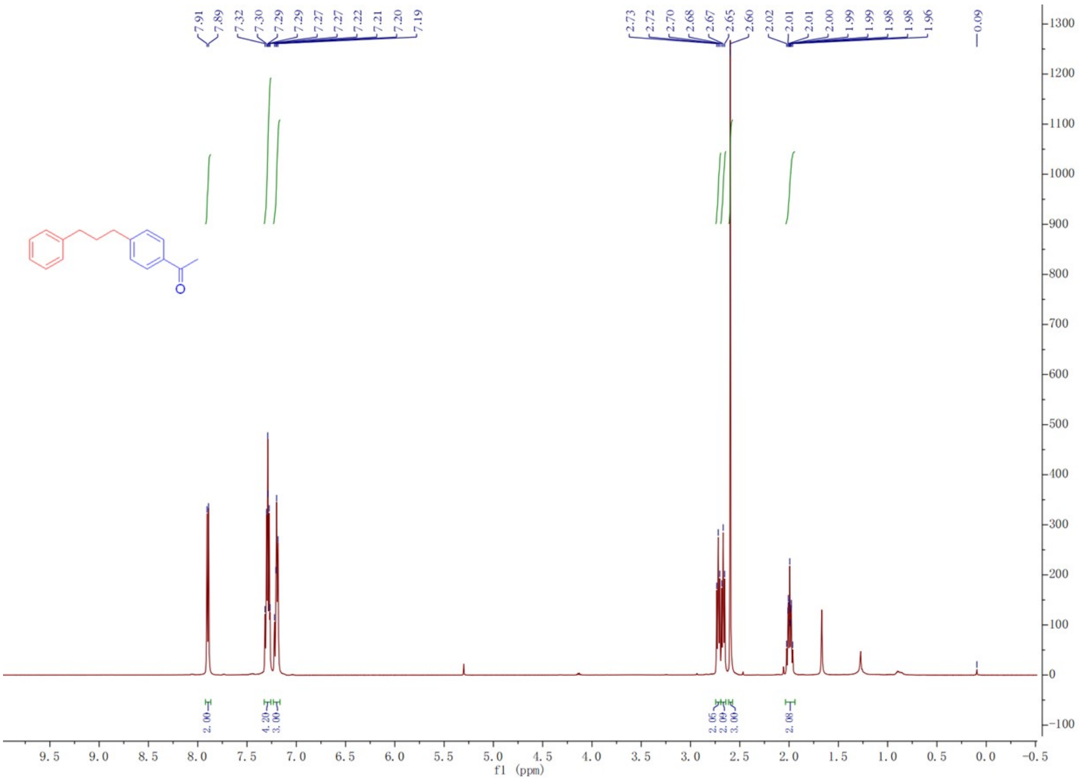

^{13}C NMR spectra of 1-(4-(3-phenylpropyl)phenyl)ethan-1-one 3g

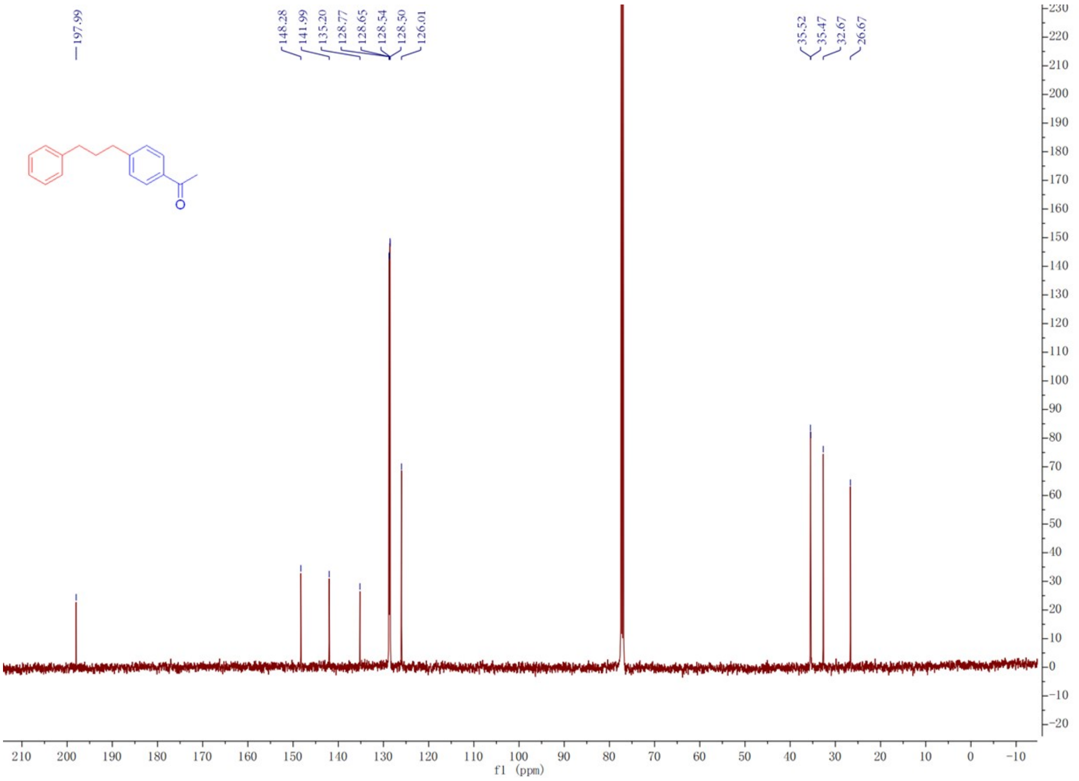

^{1}H NMR spectra of phenyl(4-(3-phenylpropyl)phenyl)methanone 3h

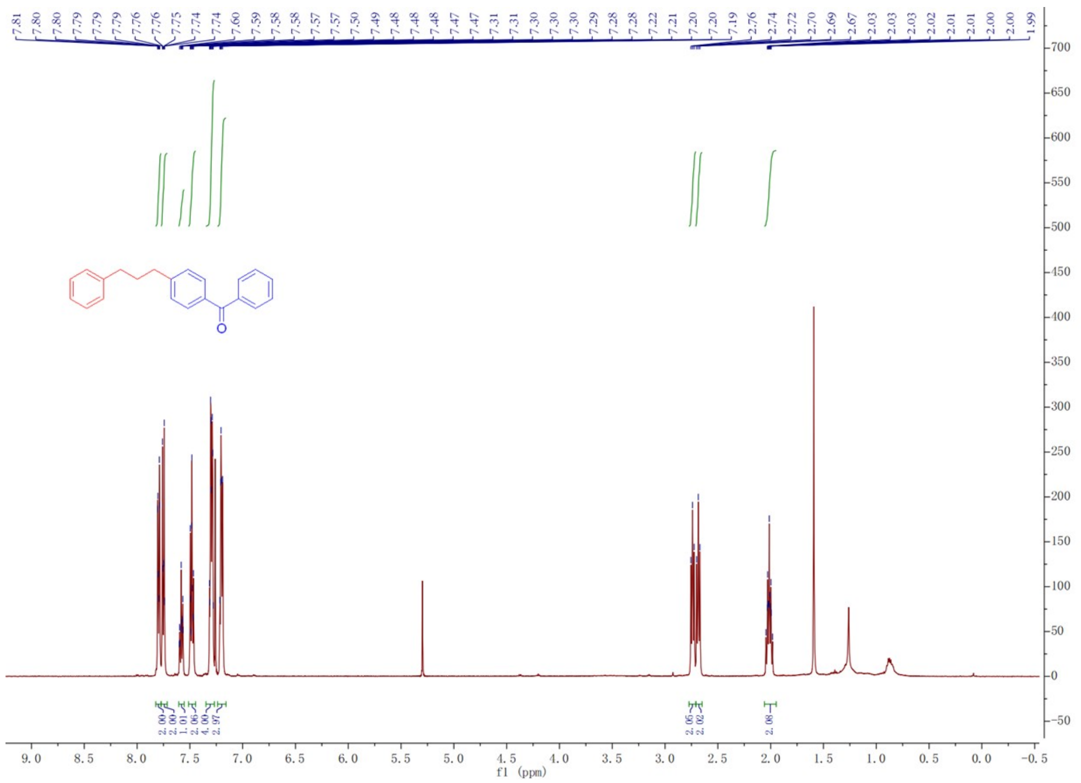

^{13}C NMR spectra of phenyl(4-(3-phenylpropyl)phenyl)methanone 3h

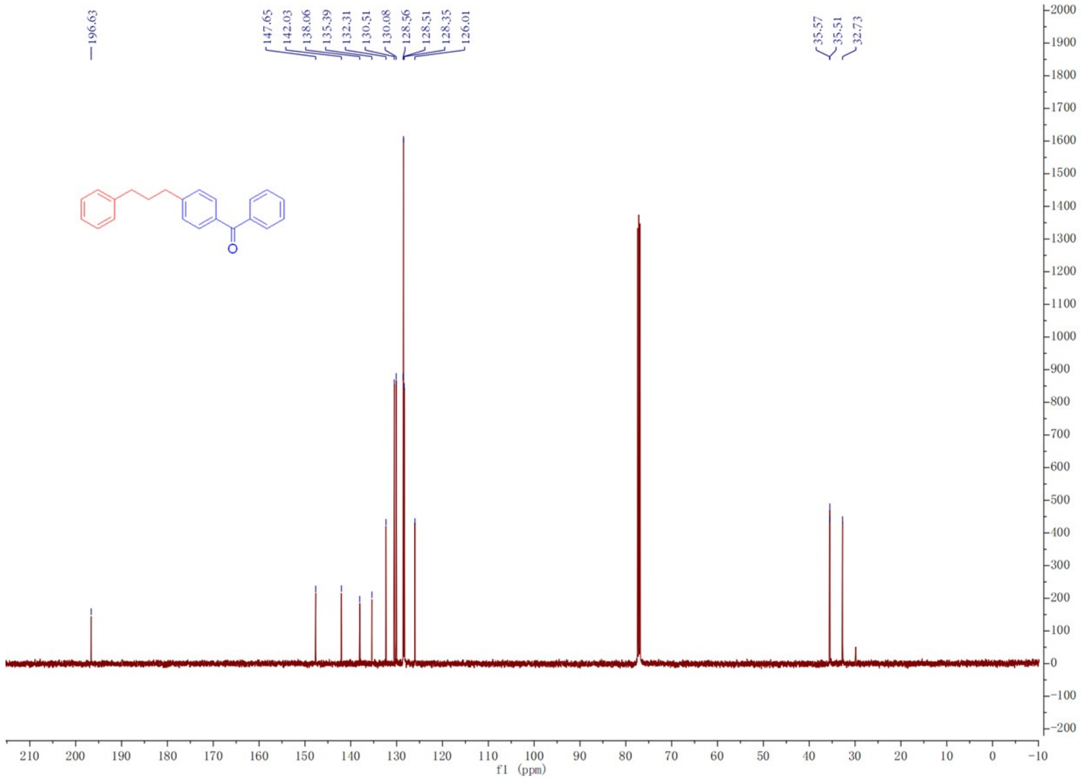

^{1}H NMR spectra of 4-(3-phenylpropyl)-1,1'-biphenyl 3i

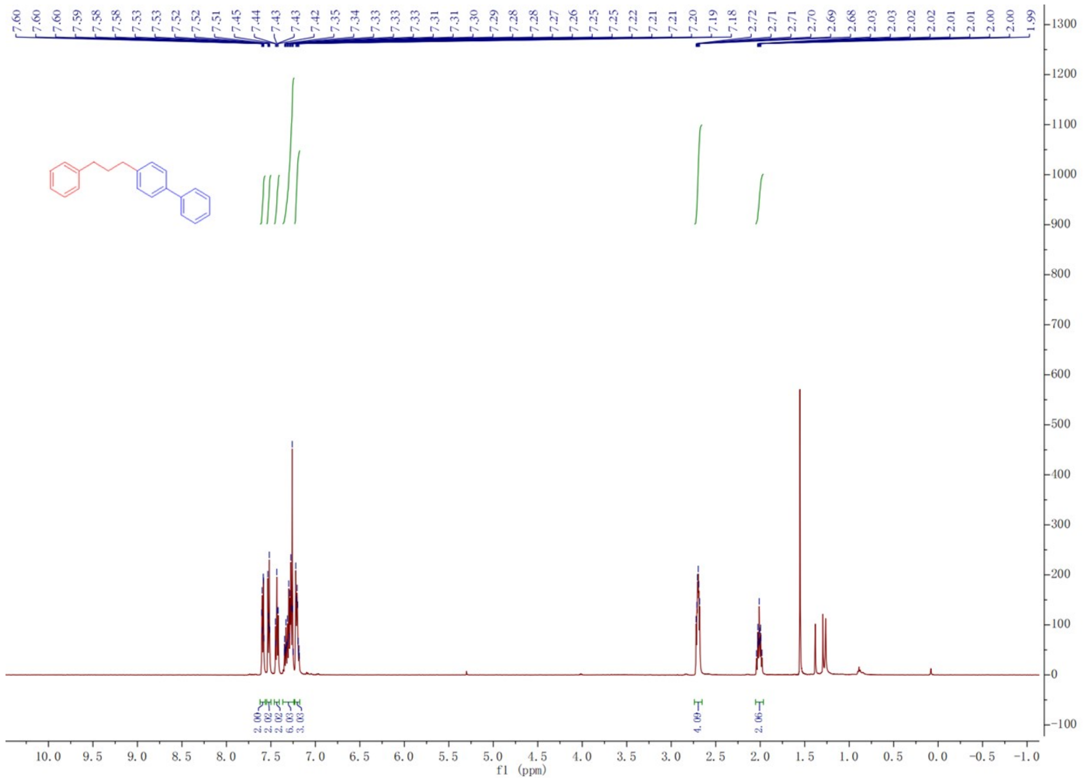

^{13}C NMR spectra of 4-(3-phenylpropyl)-1,1'-biphenyl 3i

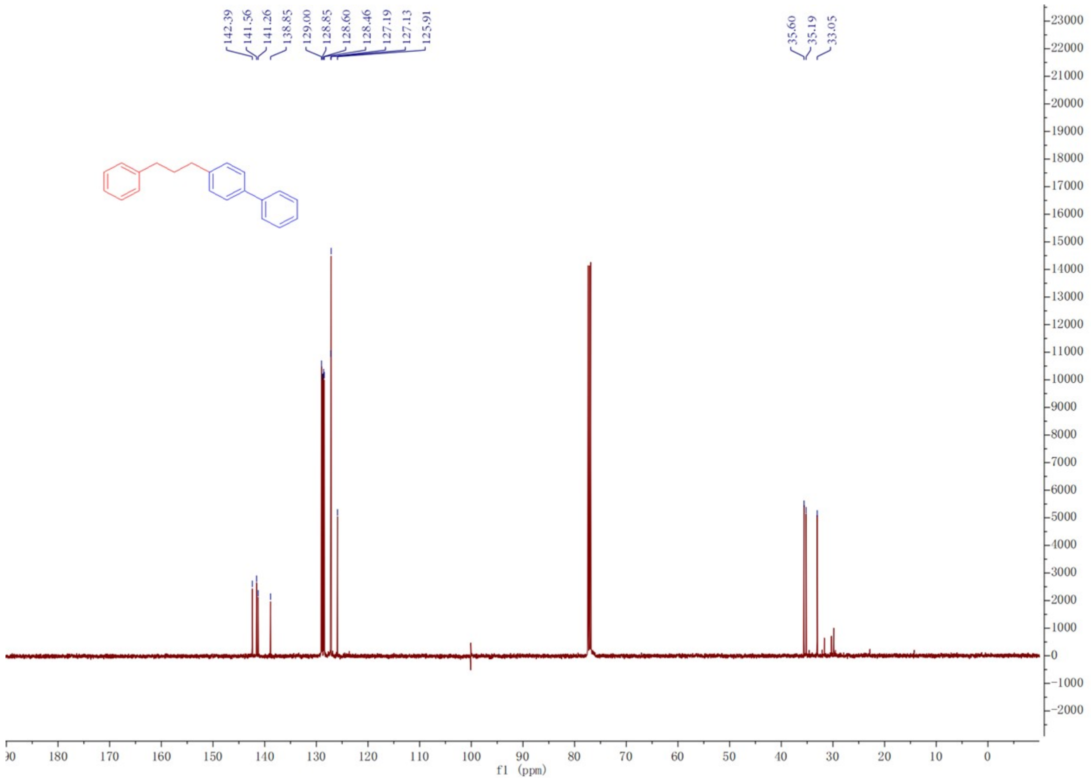

^{1}H NMR spectra of ethyl 4-(2-phenoxyethyl)benzoate 3j

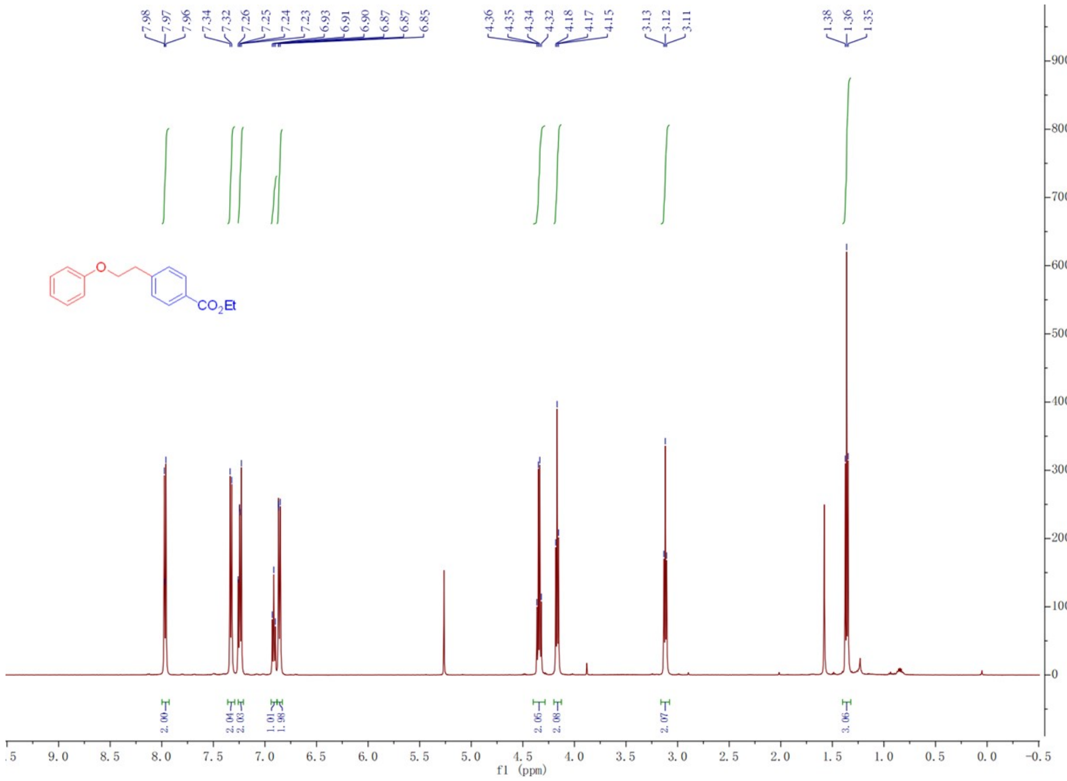

^{13}C NMR spectra of ethyl 4-(2-phenoxyethyl)benzoate 3j

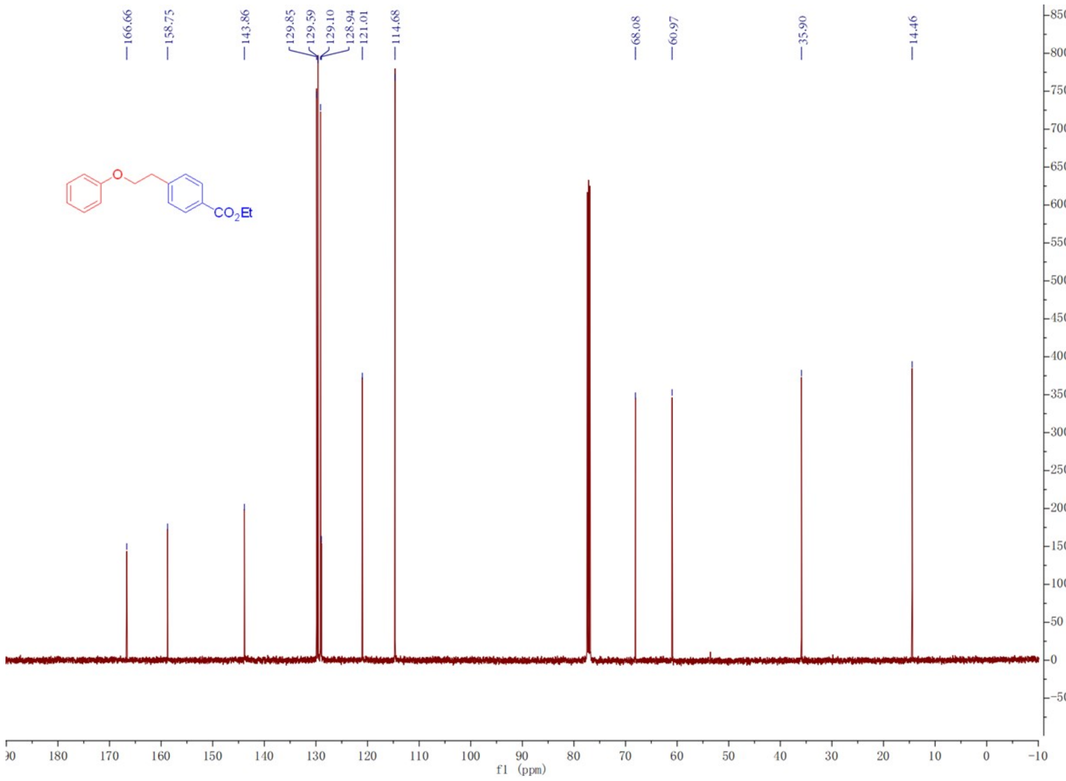

^{1}H NMR spectra of ethyl 4-(3-(benzyloxy)propyl)benzoate 3k

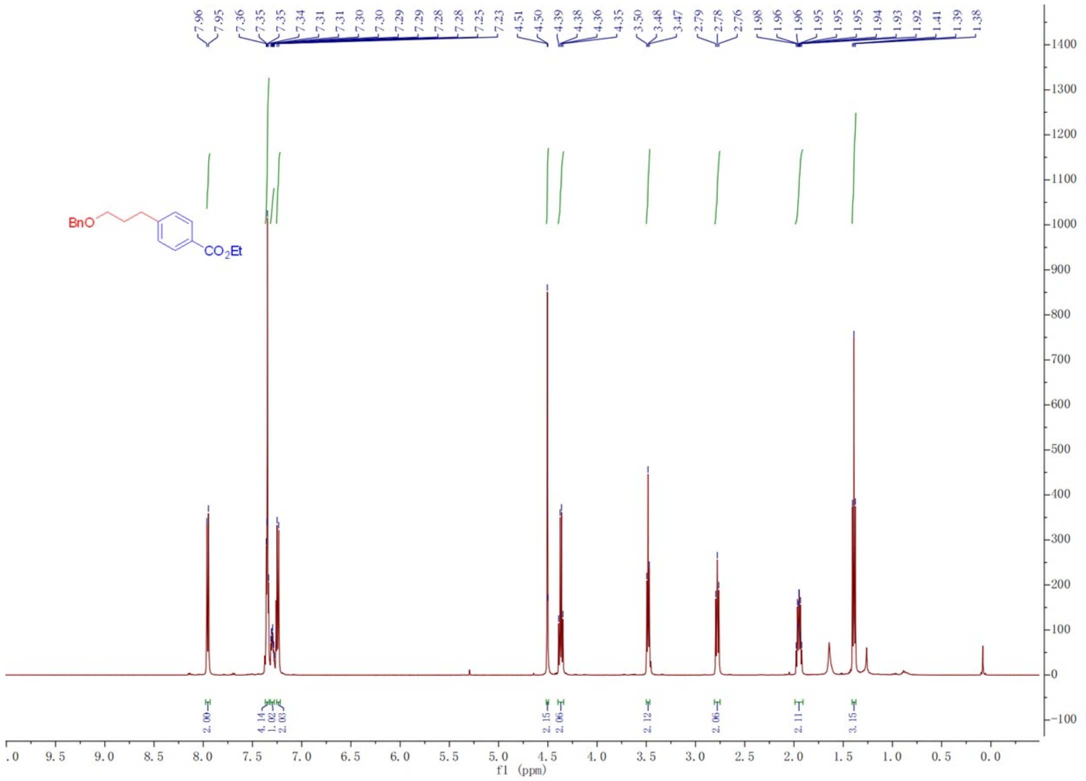

^{13}C NMR spectra of ethyl 4-(3-(benzyloxy)propyl)benzoate 3k

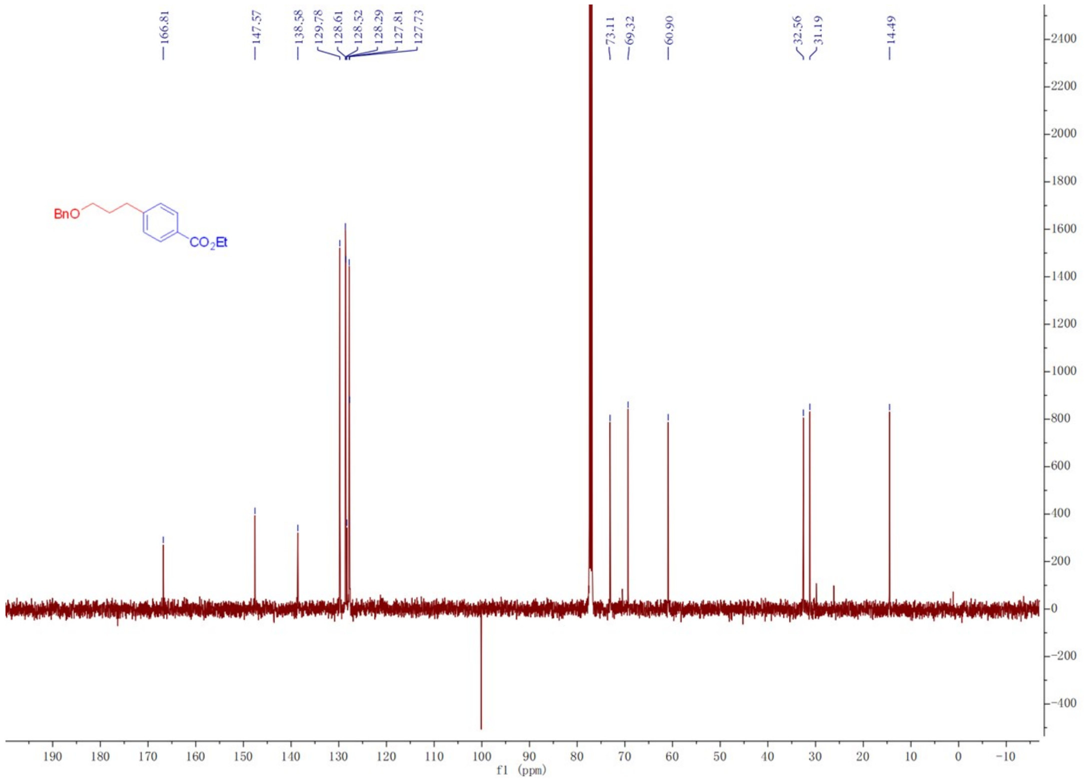

^{1}H NMR spectra of ethyl 4-(2-(1,3-dioxoisoindolin-2-yl)propyl)benzoate 3l

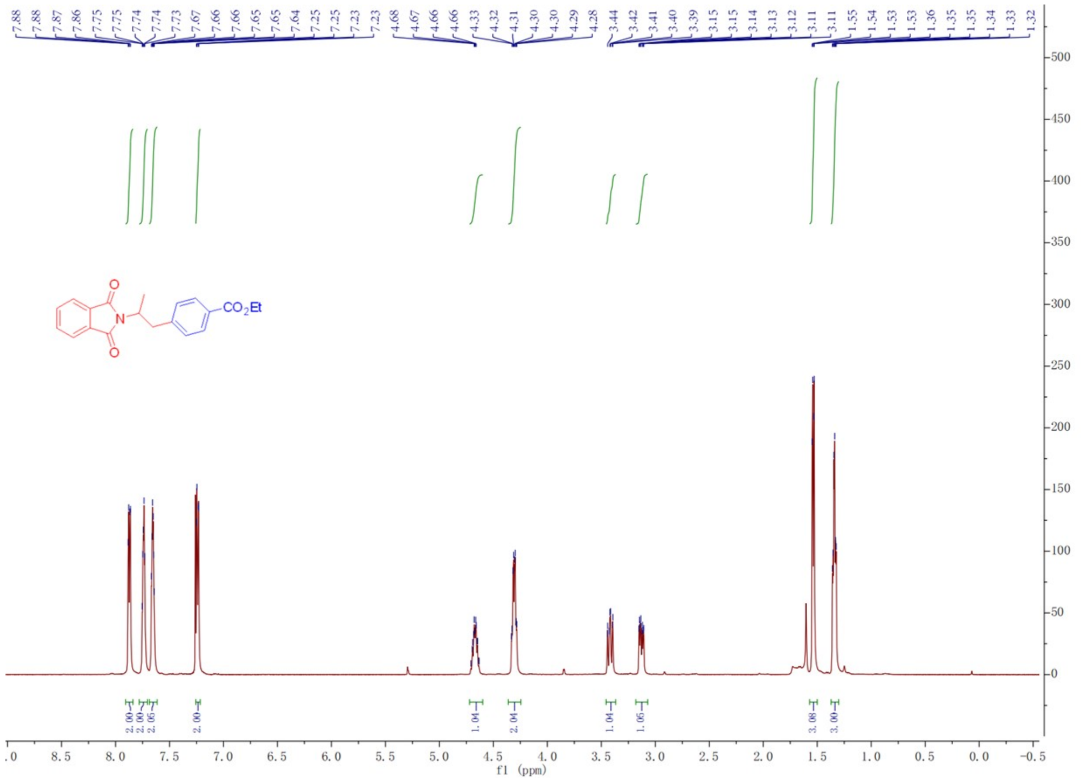

^{13}C NMR spectra of ethyl 4-(2-(1,3-dioxoisoindolin-2-yl)propyl)benzoate 3l

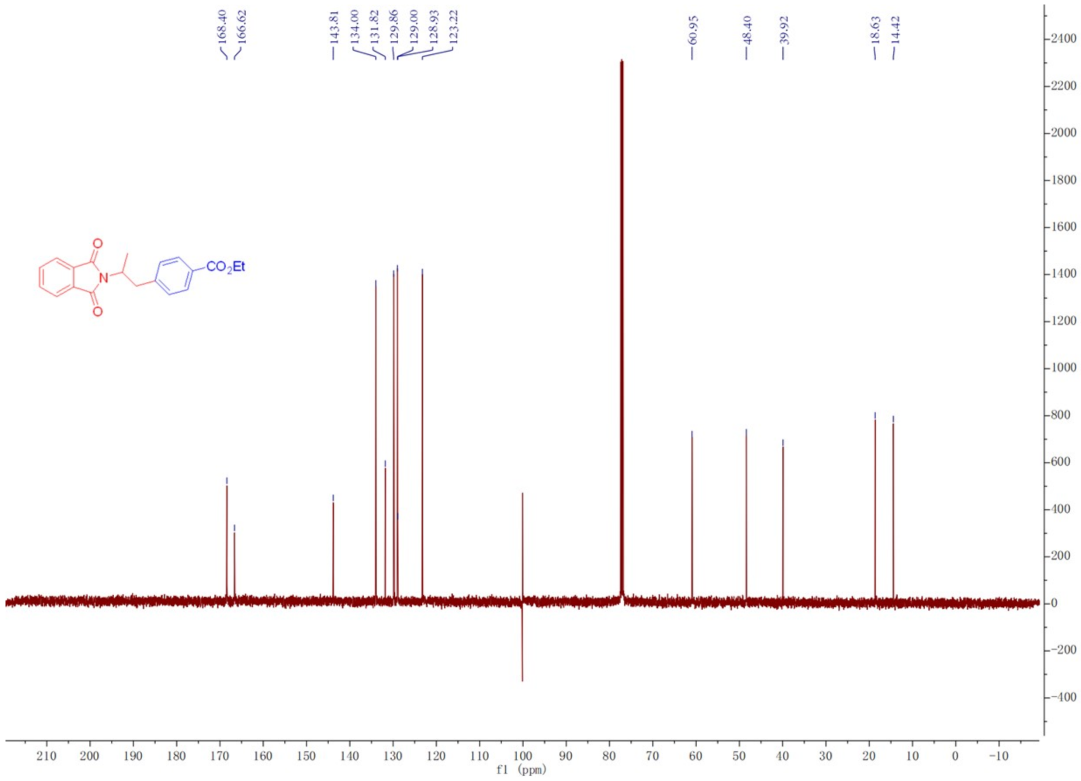

^{1}H NMR spectra of ectra of ethyl 4-(2-(1,3-dioxoisoindolin-2-yl)ethyl)benzoate 3m

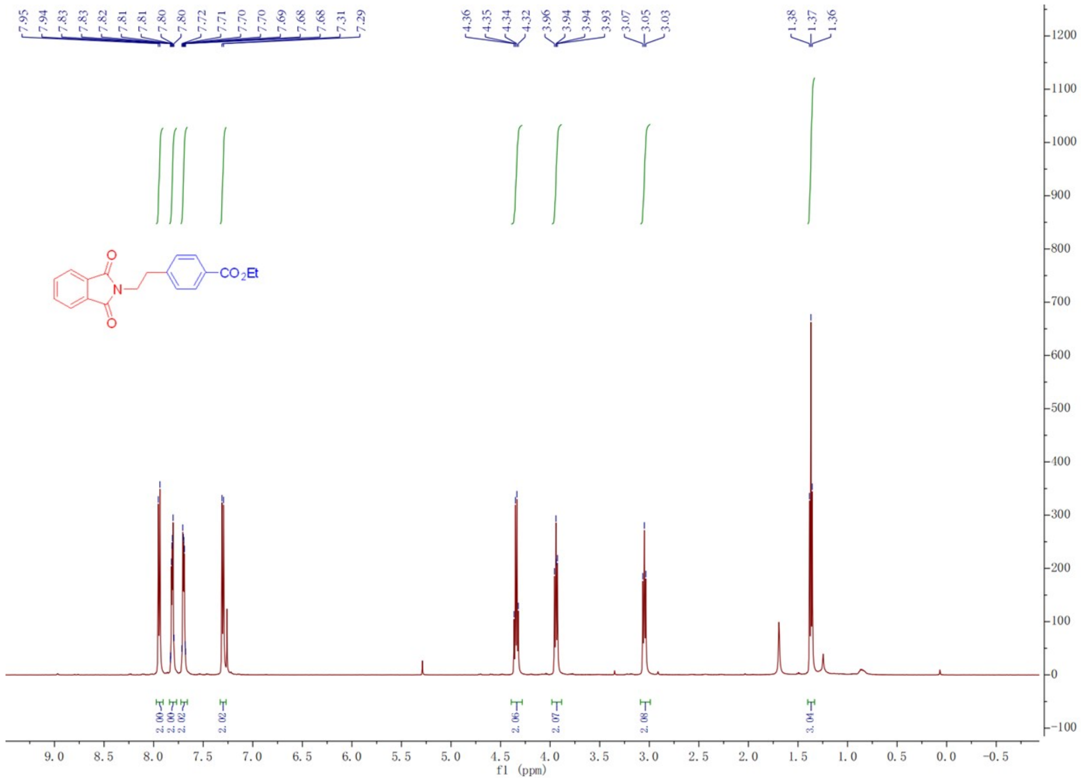

^{13}C NMR spectra of ethyl 4-(2-(1,3-dioxoisoindolin-2-yl)ethyl)benzoate 3m

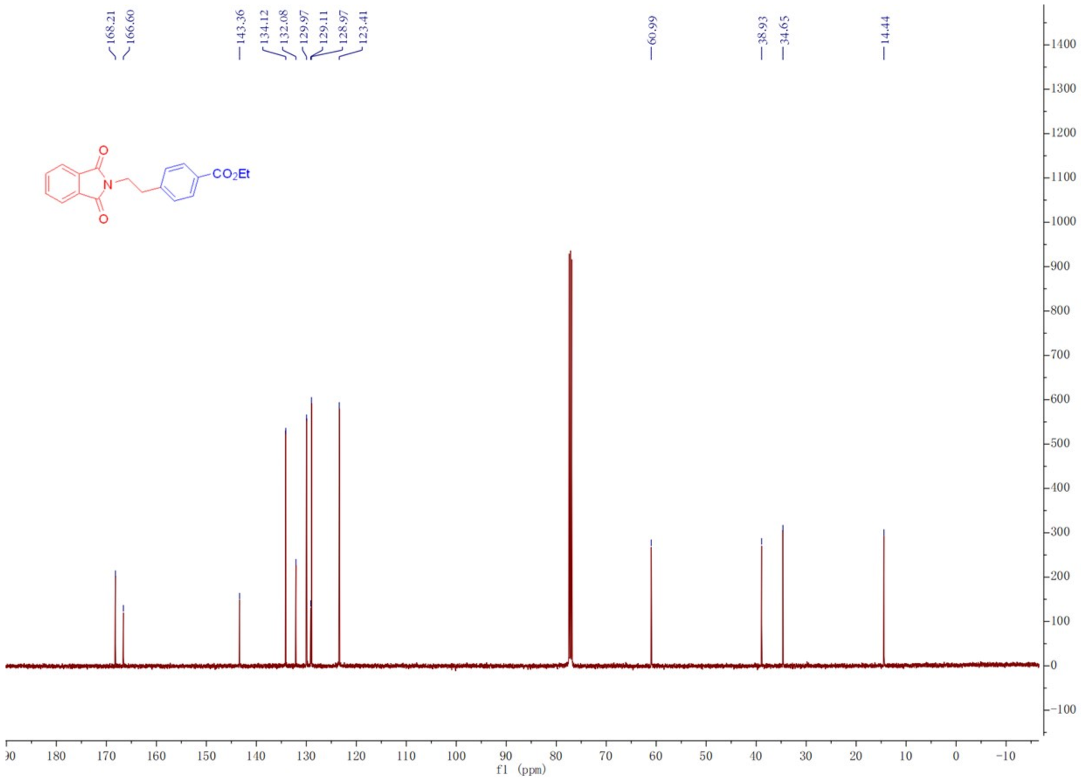

^{1}H NMR spectra of ethyl 4-(4-phenylbutyl)benzoate 3n

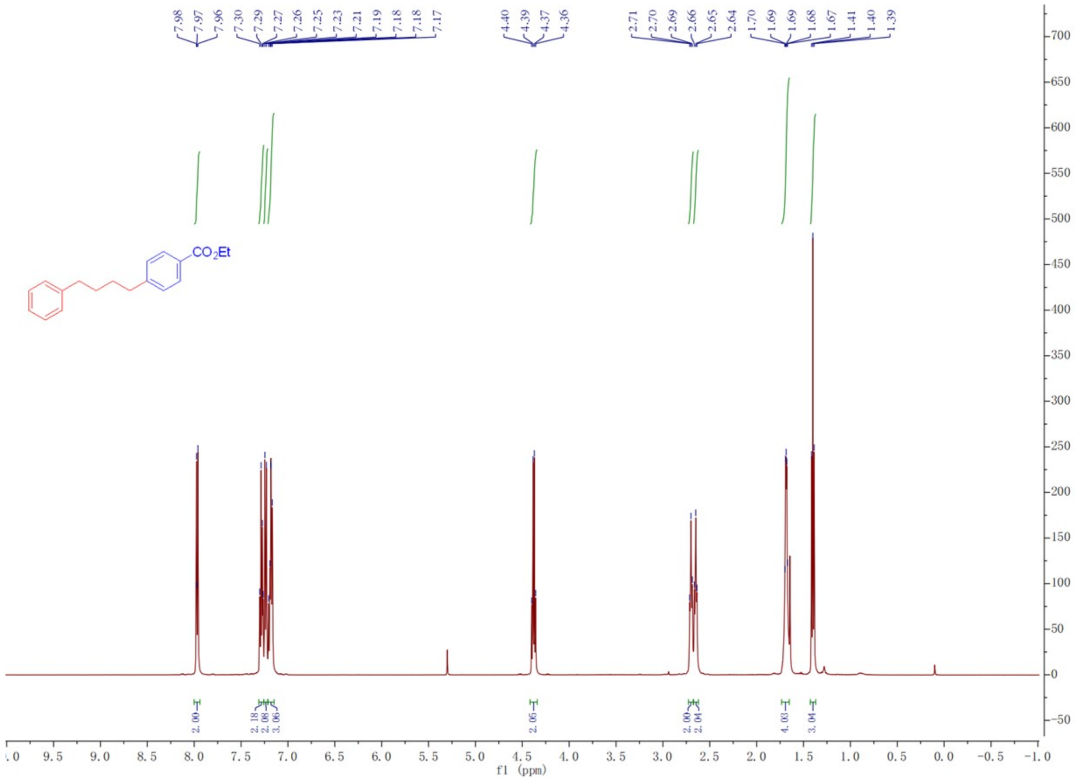

^{13}C NMR spectra of ethyl 4-(4-phenylbutyl)benzoate 3n

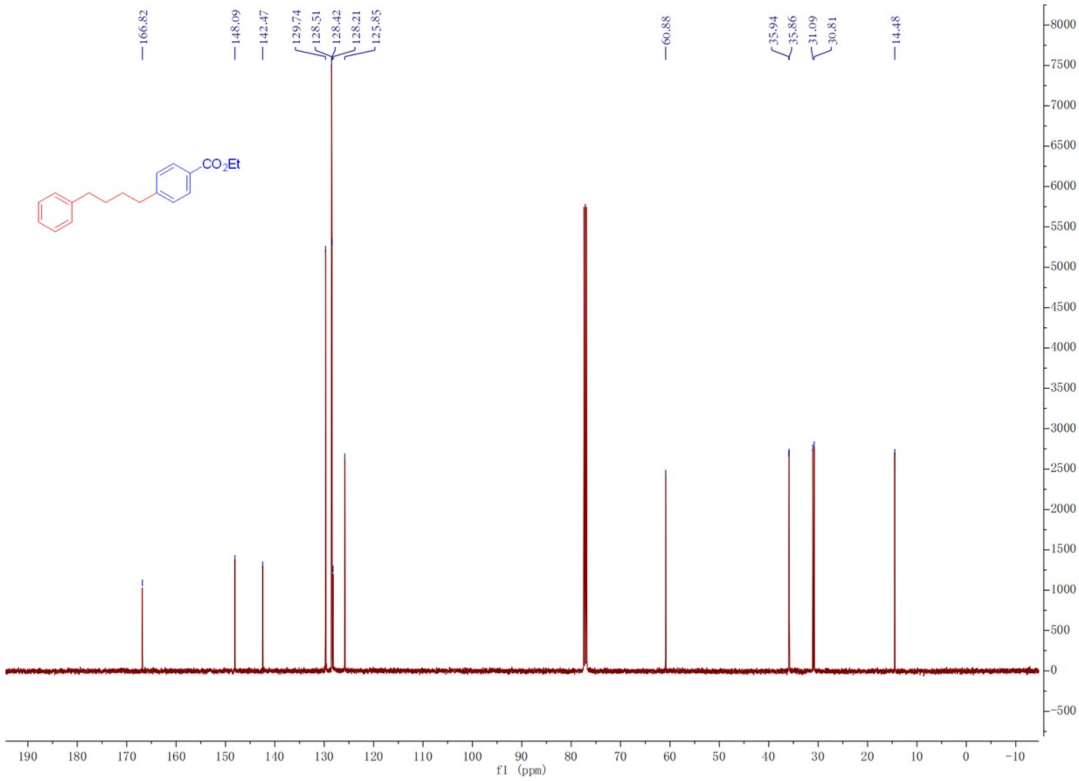

^{1}H NMR spectra of ethyl 4-phenethylbenzoate 3o

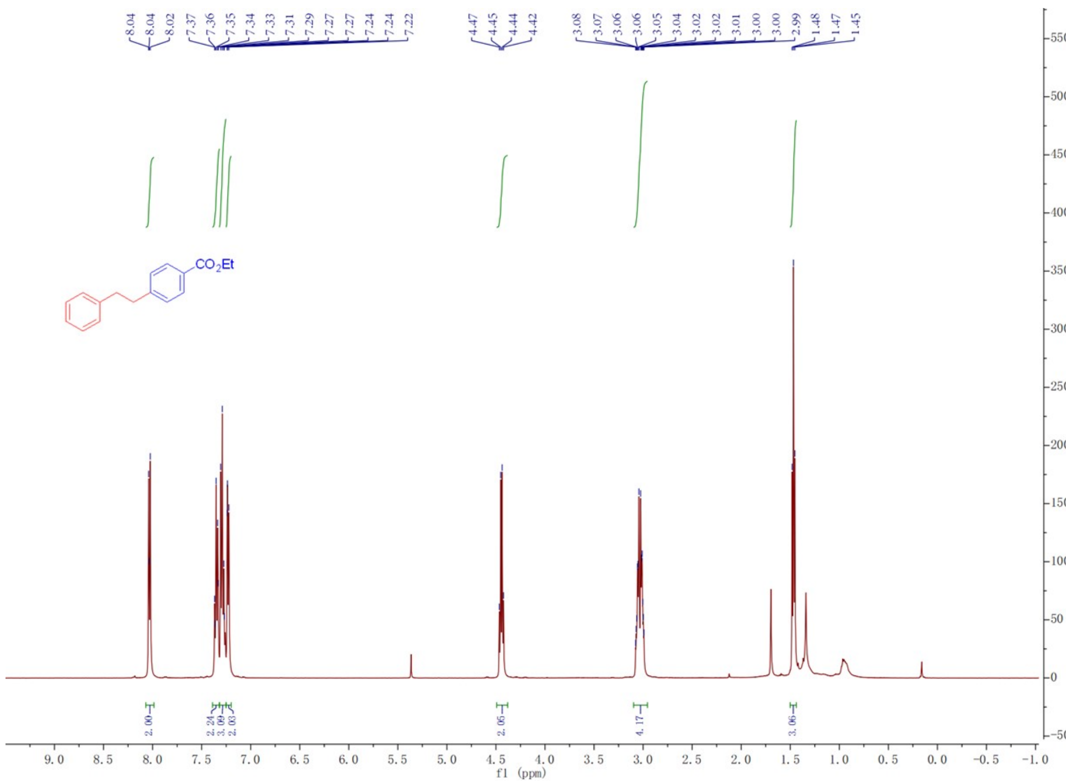

^{13}C NMR spectra of ethyl 4-phenethylbenzoate 3o

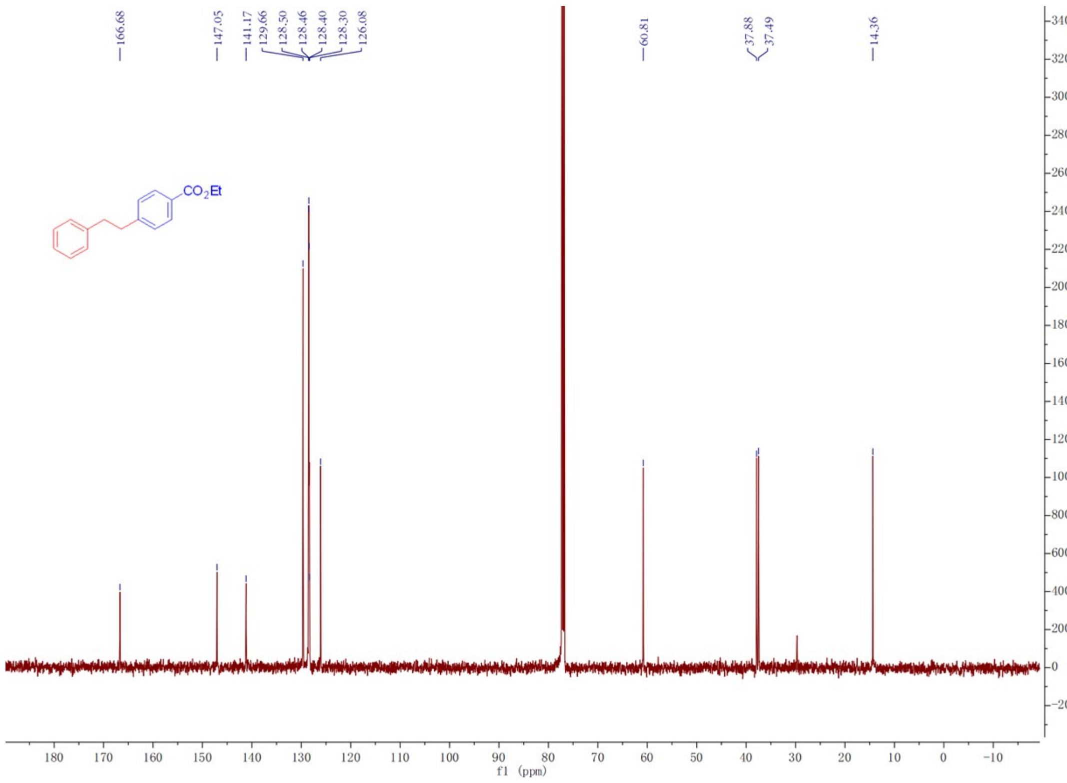

Supplement: RA-012-D2RA00010E-s001 [file RA-012-D2RA00010E-s001.pdf]
